# Reelin Controls Progenitor Cell Migration in the Healthy and Pathological Adult Mouse Brain

**DOI:** 10.1371/journal.pone.0020430

**Published:** 2011-05-27

**Authors:** Sandrine Courtès, Julien Vernerey, Lluís Pujadas, Karine Magalon, Harold Cremer, Eduardo Soriano, Pascale Durbec, Myriam Cayre

**Affiliations:** 1 Institut de Biologie du Développement de Marseille Luminy, CNRS, Université de la Méditerranée, Marseille, France; 2 Institute for Research in Biomedicine, Barcelona, Centro de Investigación en Red sobre Endfermedades Neurodegenerativas, and Department of Cell Biology, University of Barcelona, Barcelona, Spain; Instituto de Medicina Molecular, Portugal

## Abstract

Understanding the signals that control migration of neural progenitor cells in the adult brain may provide new therapeutic opportunities. Reelin is best known for its role in regulating cell migration during brain development, but we now demonstrate a novel function for reelin in the injured adult brain. First, we show that Reelin is upregulated around lesions. Second, experimentally increasing Reelin expression levels in healthy mouse brain leads to a change in the migratory behavior of subventricular zone-derived progenitors, triggering them to leave the rostral migratory stream (RMS) to which they are normally restricted during their migration to the olfactory bulb. Third, we reveal that Reelin increases endogenous progenitor cell dispersal in periventricular structures independently of any chemoattraction but via cell detachment and chemokinetic action, and thereby potentiates spontaneous cell recruitment to demyelination lesions in the corpus callosum. Conversely, animals lacking Reelin signaling exhibit reduced endogenous progenitor recruitment at the lesion site. Altogether, these results demonstrate that beyond its known role during brain development, Reelin is a key player in post-lesional cell migration in the adult brain. Finally our findings provide proof of concept that allowing progenitors to escape from the RMS is a potential therapeutic approach to promote myelin repair.

## Introduction

Cell migration is a critical aspect of brain development as it is required for correct positioning of specific cell populations. In contrast, cell migration in the adult brain is quite limited. One notable exception is the rostral migratory stream (RMS). In rodents, this pathway constitutes the highway for subventricular zone (SVZ)-derived neural progenitors en route to the olfactory bulb (OB). Tangential homophilic chain migration is a hallmark of cell migration in the RMS [1]. Migrating progenitors, often following blood vessels [2], are surrounded by a tunnel of astrocytes [2], [3], which facilitate progenitor cell migration in an adult environment and contribute to restricting them to the RMS.

Pathological conditions such as brain lesions, lead to glial or neuronal degeneration, and replacing these lost cells is a major challenge. The brain is known to activate a post-lesional plasticity program that include the mobilization of endogenous neural stem /progenitor cells (NSPCs), and their rerouting towards inflamed areas [4], [5], [6], [7], [8], [9]. However, these spontaneous repair attempts are limited and often not efficient enough to achieve functional recovery. Understanding the mechanisms controlling such processes represent a key step to promote brain repair. To date, most studies that attempted to favor endogenous repair have been devoted to growth factors [9]. Here, we propose to test a new promising strategy targeting migration behavior of SVZ progenitors that could be efficient to facilitate endogenous cell recruitment to the lesions.

Reelin is a secreted extracellular matrix protein playing a crucial role in neuroblast migration and positioning in the developing brain, notably for proper cortical lamination [10]. In the adult brain, although its expression is strongly down regulated, Reelin is maintained in few structures, notably in the OB where it acts as a detachment signal on migrating neuroblasts by changing their migration mode from cooperative tangential to individual radial migration [11], thus allowing their integration into the OB.

Here, we uncover a new role for Reelin in post-lesional cell migration in the adult brain and provide proof of concept that allowing cells to escape from the RMS promotes endogenous cell replacement at the lesion site in a mouse model of demyelination.

## Results

### Reelin is endogenously up-regulated after brain lesion

**Figure 1:**
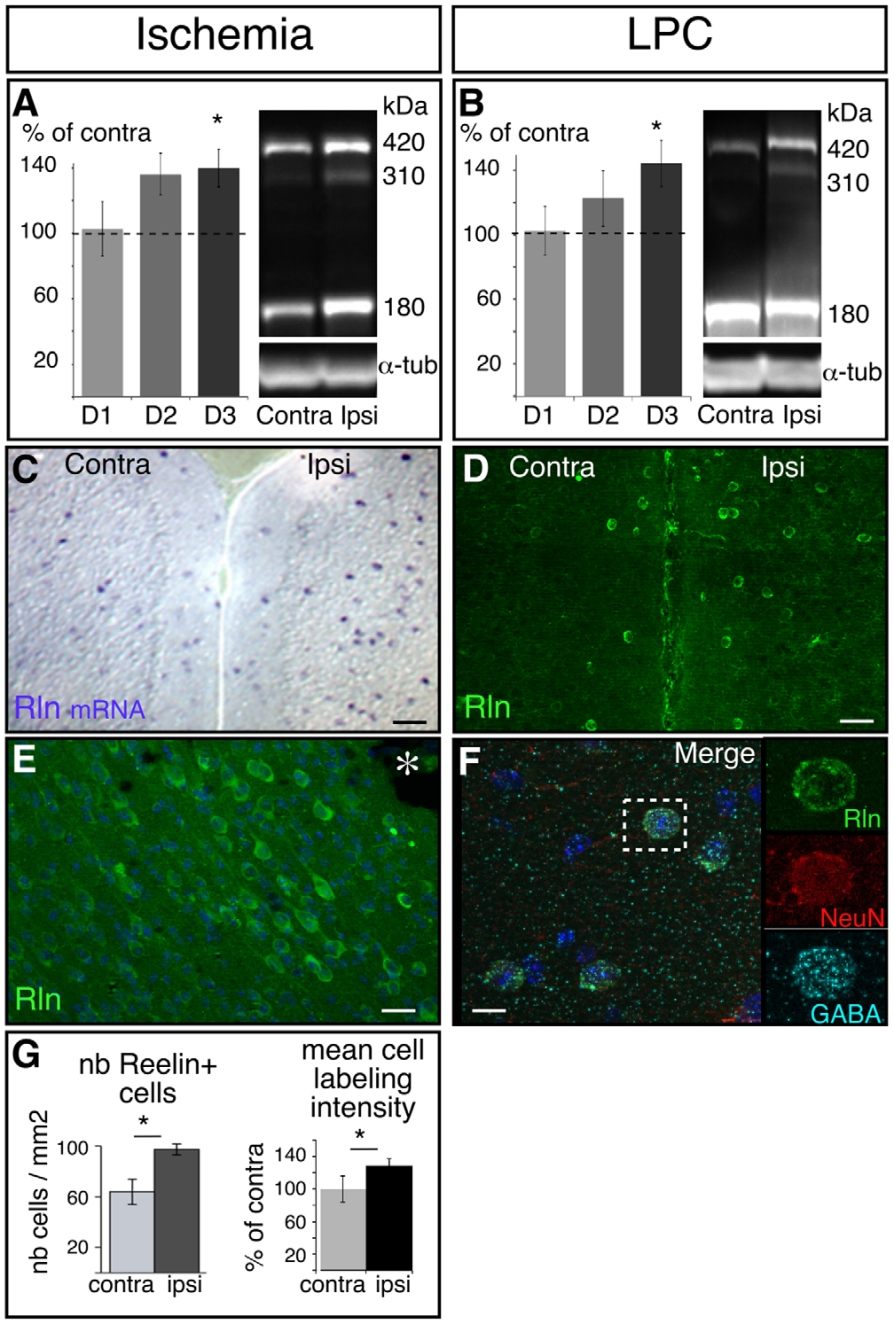
Reelin is up-regulated in the periphery of the lesion in two different lesion models. A–B: Western blot and semi-quantitative analysis of Reelin expression in peri-lesional areas 1 to 3 days (D1, D2, D3) after unilateral cortical ischemia (A) or demyelination (B). Reelin expression was normalized to α-tubulin and expressed in percentage of contralateral (unlesioned) side (from 5 or 6 mice). Western blot illustrations in A and B are representative of Reelin expression on day 3. C, D: In situ hybridization (C) and Reelin immunolabeling (D) in the cortex 3 days after lesion revealing higher number of labeled cells in the ipsilateral cortex. E: Cell immunoreactivity is maximal in close proximity to the lesion. The asterisk (*) indicates the core of the thermocoagulation lesion site. F: Reelin-positive cells (green) colocalize with NeuN (red) and GABA-positive (light blue) interneurons in the cortex after demyelination lesion. G: Quantification of Reelin expressing cells (in situ hybridization) and of labeling intensity (immunolabeling) in cortex contralateral or proximal to a thermocoagulation lesion. contra: areas from contralateral side to the lesion; ipsi: areas proximal to the lesion; LPC: lysolecithine demyelinating lesion. Scale bars: C = 100 µm; D = 50 µm; E = 25 µm; F = 10 µm.

We first questioned whether endogenous Reelin expression could be modulated in response to brain injury. We used the two following lesion models: lysolecithin (LPC) induced focal demyelination of the corpus callosum, and unilateral cortical ischemia induced by thermocoagulation of pial arterioles, a model in which alterations in other extracellular matrix proteins are suggested to have a prominent role [12]. Using semi-quantitative Western blot analysis, we found a rapid (after thermocoagulation) or progressive (after LPC) increase in Reelin expression in the perilesional structures in the ipsilateral compared to the contralateral side (Fig. 1A,B). It reached significance 3 days after lesion when Reelin expression was increased by 40% in thermocoagulated cortex (Fig. 1A) and by 44% in the structures adjacent to demyelination lesions (Fig. 1B) (p<0.05, n = 5 in both cases).

To determine the origin of the source of Reelin, we performed in situ hybridization (Fig. 1C) and immunofluorescence (Fig. 1D–F). In both lesion models we detected numerous Reelin expressing cells in the cortex, in perilesional area but not in the core of the lesion. However, in the ischemia model, the highest number of cells with high Reelin expression was in close proximity to the infarct (Fig. 1E). After demyelination lesions, Reelin-expressing cells were also restricted to the cortex and not observed inside the corpus callosum (Fig. 1D).

Three days after ischemia, the number of cells expressing Reelin mRNA was significantly higher in the cortex ipsilateral to the lesion than in the contralateral cortex (97.3±4.5 vs. 64.0±9.9 cells/mm^2^ respectively, p<0.05, n = 3); furthermore, the mean labeling intensity of immunopositive cells was significantly increased by 29% in the former compared to the latter (p<0.05, n = 4) (Fig. 1G). This reactivity was still present 7 days after lesion, although weaker (data not shown).

Using co-immunolabeling 3 days post-lesion in the two models, we showed that the cells responsible for Reelin overexpression were not glia (GFAP-negative and Olig2-negative), microglia or macrophage (IΒ4-negative) or infiltrated blood cells (CD3-negative) (Figure S1). As demonstrated by NeuN co-expression (Fig. 1F), these Reelin+ cells belonged to the neuronal cell population, and almost all of them appeared to be GABAergic interneurons (Fig. 1F). In conclusion, in response to brain damage, Reelin expression is reactivated in mature neurons in proximal areas. The reactivation of a developmental cue in a lesional context raises the question as to the role of Reelin in post-lesional plasticity.

### Reelin overexpression promotes SVZ-derived NSPC dispersion in periventricular structures in vivo

To study whether Reelin could influence NSPC migration in a mature brain, we ectopically grafted Reelin-expressing cells in adult healthy brains and examined the effect of this ectopic source of Reelin on the migratory behavior of SVZ-derived cells.

**Figure 2:**
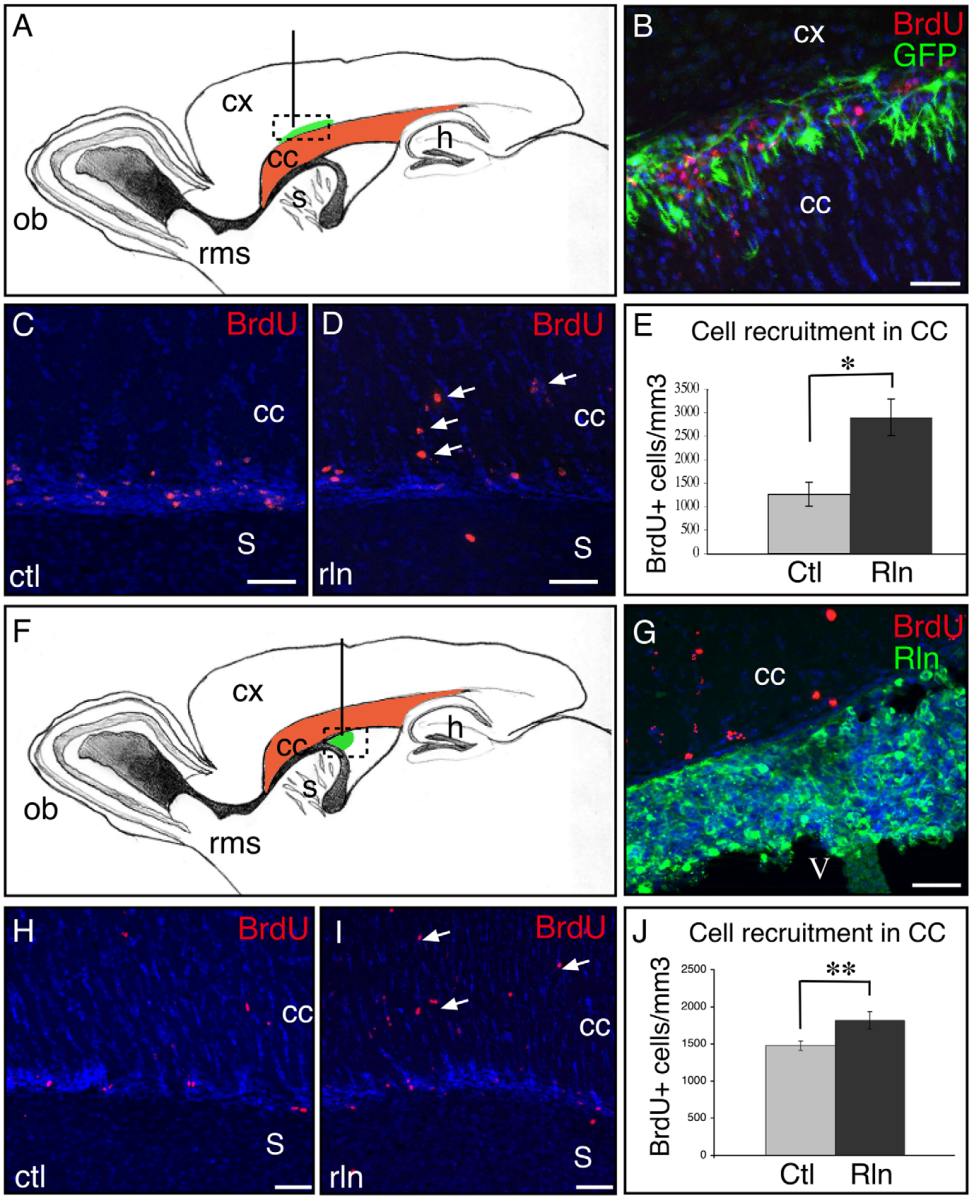
Graft of Reelin-expressing cells increases the recruitment of SVZ-derived cells toward sub cortical white matter tracts. A,B: Illustration of the migration of grafted NSPCs along the cingulum (in green) and BrdU-labeled cells (in red) that migrated from the SVZ. C,D and H,I: SVZ-derived BrdU-labeled cells migrate along the RMS and are recruited in the CC (arrows) of mice grafted with control (C,H) or Reelin-secreting cells (D,I). E: Quantification of SVZ-derived BrdU-labeled cells recruited in the corpus callosum 7 days after NSPC graft in the cingulum. F,G: Illustration of HEK cells secreting Reelin grafted in the ventricle. J: Quantification of SVZ-derived cells recruited in the CC 7 days after HEK cell graft in the ventricle. Reelin increases the recruitment of SVZ-derived cells toward CC independently of the location of the Reelin source (in the corpus callosum (A–E) or in the ventricle (F–J)). CC: corpus callosum; Cx: cortex; s: striatum. V: ventricle. Scale bars  = 50 µm.

We first used a primary culture of nenonate neural progenitors from actin-GFP mice, grown as neurospheres and transiently nucleofected to express Reelin (NS-Rln) or DsRed for controls (NS-DsRed). Western blot analysis of cell supernatant verified that Reelin was indeed produced and secreted by NS-Rln cells at the time of implantation whereas it was undetectable in NS-DsRed medium (Figure S2D). In order to obtain Reelin expression in periventricular structures, we grafted these genetically modified neurospheres into the subcortical fiber tracts (Fig. 2A), generating a ribbon of Reelin-expressing cells in and above the corpus callosum (Fig. 2B and Figure S2B). Endogenous SVZ-derived NSPCs were traced with BrdU (see methods), and cell dispersion was estimated by counting the number of SVZ-derived cells that escaped the RMS to enter the corpus callosum. The number of BrdU+ cells/mm^3^ found in the corpus callosum was significantly more than doubled in NS-Rln compared to NS-DsRed grafted mice (2897±193 vs. 1261±126 cells/mm^3^, p<0.05, n = 4) (Fig. 2C–E).

In a complementary approach, we grafted HEK193 cell lines stably transfected to secrete Reelin (HEK-Rln, Figure S2D) or to express DsRed for controls (HEK-DsRed) in the lateral ventricle of nude mice (Fig. 2F). Grafted HEK cells grew quickly in the ventricle (Fig. 2G), generating a Reelin source in the cerebrospinal fluid. In the presence of HEK-Rln graft, 23% more BrdU+ SVZ-derived cells were found in subcortical fiber tracts 7 days after grafting (p<0.01, n = 4) (Fig. 2H–J). The number of BrdU+ cells in the striatum was also enhanced by 30% but the difference did not reach significance (data not shown, p = 0.12, n = 4).

In conclusion, both models showed that ectopic expression of Reelin in the vicinity of the SVZ favors the emigration of NSPCs from the SVZ/RMS and allows their dispersion toward adjacent structures, independently of the precise localization of the Reelin source.

### Transgenic mice overexpressing Reelin in the adult forebrain show RMS defects with altered cell migration

**Figure 3:**
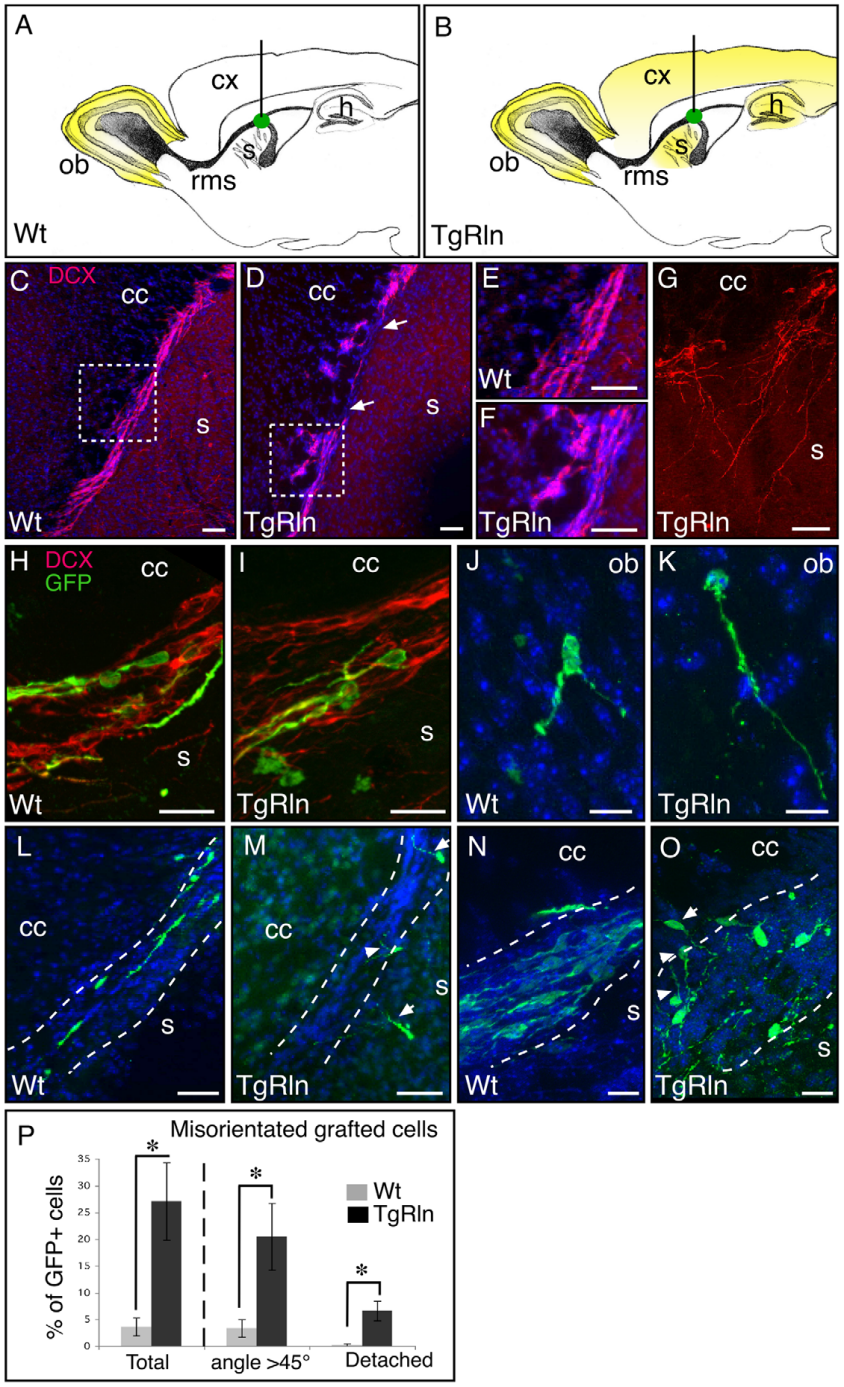
Cell migration in the RMS is altered in transgenic mice overexpressing Reelin in the forebrain. The overall RMS structure was revealed by DCX immunofluorescence, and individual cell migration in the RMS was analyzed after graft of GFP+ SVZ cells into the SVZ-RMS corner. A,B: Schematic representation of the graft of GFP+ SVZ cells (in green) in the SVZ/RMS of Wt (A) and transgenic mice overexpressing Reelin (B) (Reelin expression is schematized in yellow). C–G: Migrating neuroblasts (DCX+, in red) in the RMS of Wt (C,E) and TgRln (D,F,G) mice. E and F are enlarged views of the white squares in C and D respectively. Note the disorganization of the RMS in transgenic mice, with interruptions in the RMS (between arrows in D), and neuroblasts escaping the RMS in chains (F) or individually (G) and showing misoriented processes and a complex morphology. H,I: GFP+ SVZ grafted cells (in green) integrate and migrate in the RMS (DCX in red) of control (H) and TgRln (I) mice. J,K: GFP+ SVZ cells reached the olfactory bulb 7 days after grafting in Wt (J) as in TgRln (K) mice. L–O: GFP+ grafted cells remain confined to the RMS in Wt mice (L,N) whereas they tend to be misoriented (arrowheads) or even leave the RMS (arrows) in TgRln mice (M,O). P: Quantification of the proportion of grafted cells that were misoriented (presenting an angle superior to 45° with RMS orientation) or that detached and left the RMS. CC: corpus callosum; ob: olfactory bulb; s: striatum; TgRln: transgenic mice overexpressing Reelin; Wt: Wild-type mice. Scale bars : C, D = 100 µm; E,F,G,L,M  = 50 µm; H,I,N,O  = 20 µm; J,K  = 10 µm.

In order to confirm the effect of global Reelin overexpression on SVZ cell migratory behavior, we used a conditional transgenic mouse line which over-expresses Reelin (TgRln), under the control of the CamKII promoter, in the postnatal and adult forebrain (but not during development therefore excluding developmental defects). Matching the expression of the CAMKII promoter [13], this strain shows a 3–6 fold increase in Reelin expression in the neocortex, striatum and OB compared to controls (Pujadas et al., 2010) (schematized in yellow in Fig. 3A,B). The anatomy and cellular organization of the SVZ-RMS system of these animals was examined in detail on sagittal sections. Doublecortin labeling (DCX, a well-established marker of migrating neuroblasts in adult neurogenesis, see [14]) revealed interruptions in the RMS of TgRln animals (Fig. 3D arrows) with chains of DCX+ progenitors escaping the RMS towards adjacent structures (Fig. 3D,F). Furthermore, in TgRln mice, some DCX+ cells displayed complex morphology with long and arborized processes and escaped the RMS (Fig. 3G). These experiments clearly indicate that ectopic Reelin expression induced severe disorganization of SVZ/RMS cyto-architecture. In this context, to further study individual cell migration behavior, GFP+ SVZ cells were grafted in the proximal RMS (Fig. 3A,B), and their localization and morphology were analyzed 7 to 10 days after grafting. In TgRln animals, as in Wt littermates, most grafted GFP cells integrated the RMS in which they migrated (Fig. 3H,I) to finally reach the OB (Fig. 3J,K). However, in TgRln animals only, numerous GFP cells were found with abnormal position, orientation and morphology. While in the Wt all GFP+ cells remained in the RMS with bipolar morphology (Fig. 3L,N), in TgRln animals 20% of GFP+ cells migrating in the RMS were significantly misdirected (Fig. 3M,O and P), and 7% escaped the RMS (Fig. 3M arrow and 3P). Escaped cells reached indifferently the corpus callosum (Fig. 3O and Figure S3B) or the striatum (Fig. 3M and Figure S3A) of Tg animals, showing no preferential migration toward striatum where Reelin expression is higher (as characterized in Pujadas et al., 2010). As observed with endogenous DCX+ cells, the grafted GFP cells escaped the RMS either individually or along DCX+ chains (Figure S3). Furthermore, these misoriented and detached cells had longer and more ramified processes than normal neuronal progenitors migrating in the RMS (Fig. 3G,M,O), and one third of them did not express the early markers of neuronal progenitors such as PSA-NCAM, DCX (not shown) and ßIII tubulin (Figure S3C–E), thereby suggesting that they were becoming more mature.

On the basis of these results, we conclude that Reelin is sufficient to induce the detachment of NSPCs from the RMS in vivo, thus resulting in their enhanced dispersion in periventricular structures.

### In vitro Reelin sequentially promotes SVZ-derived cell migration and neuronal progenitor maturation

**Figure 4:**
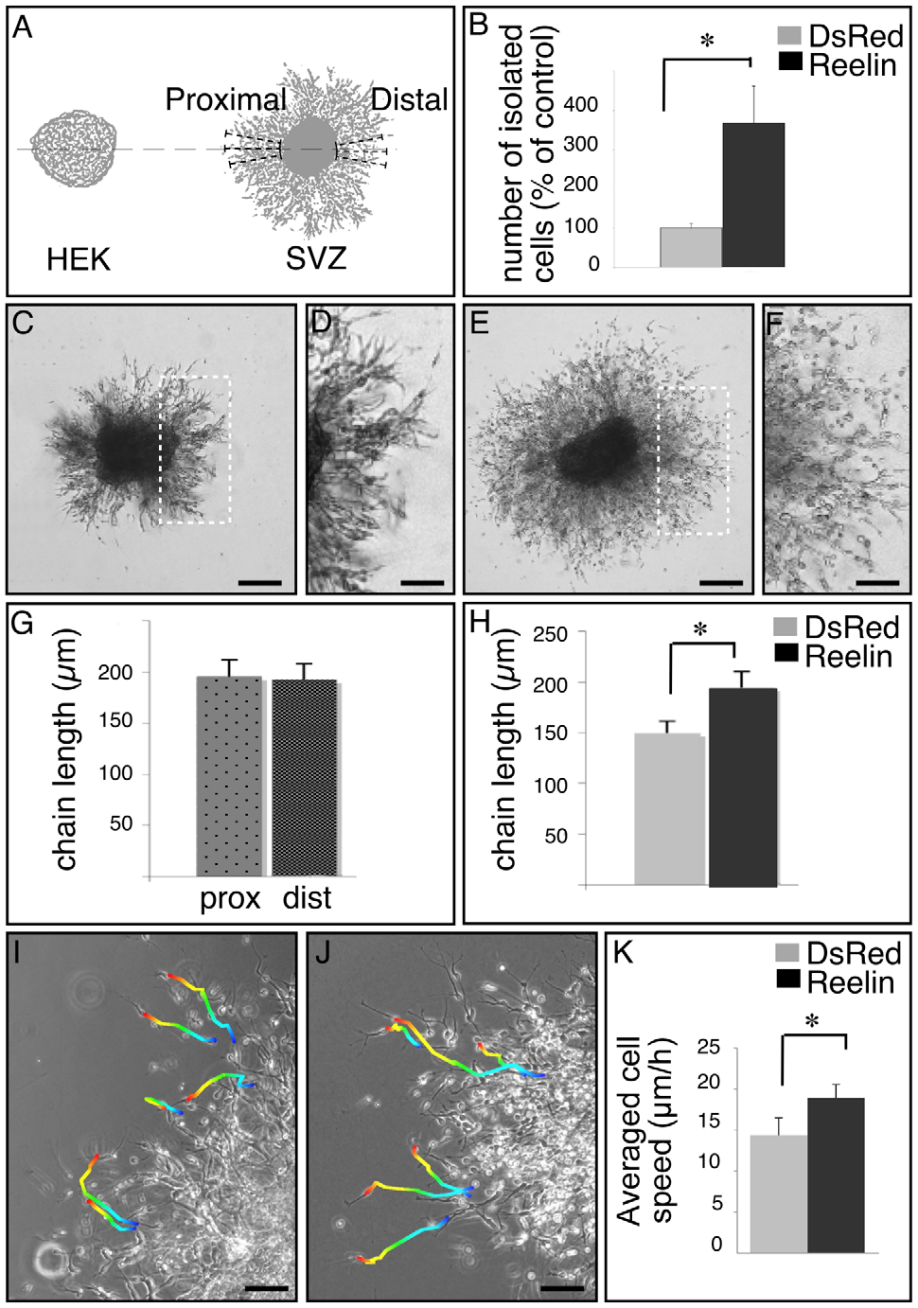
Effect of Reelin on SVZ cell migration in vitro. Reelin triggers cell detachment from the chains of migrating progenitors and increases migration speed. A: Schematic diagram of SVZ explant cultures in the presence or absence of a focal source of Reelin. Aggregates of HEK cells stably expressing Reelin (or DsRed for controls) were placed in close proximity to SVZ explants. The length of chains formed by cells migrating out of the explants was measured in 3 independent points proximally and distally to the HEK aggregate. B: Quantification of individual cells detached from the chains. C–F: Migration pattern in the absence (C,D) and presence (E,F) of Reelin. D, F: Magnification of white rectangle in C and E respectively. A large number of isolated cells migrating outside the chains can be seen in the presence of Reelin. G: Chain length proximal and distal to HEK aggregates producing Reelin. No difference can be observed between proximal and distal chain length, thereby arguing against a chemoattractant effect of Reelin. H: Chain length in Reelin versus DsRed conditions, showing a significant lengthening in the presence of Reelin. I,J: video time-lapse recording of individual migrating cells in the absence (I) or presence (J) of Reelin, with trajectory colored (blue to red along 9 h). K: Quantification of migration speed (average of more than 200 cells from 3 independent experiments). Scale bar in C,E  = 100 µm; in D,F,I,J  = 50 µm.

We used the standard in vitro migration assay of SVZ explants to study the effect of Reelin on progenitor cell migration in culture. To this end, SVZ explants (31 explants in each condition, in 5 independent experiments) were cultured in 3 dimensional Matrigel matrix, in the presence of a Reelin gradient produced by an aggregate of HEK cells expressing Reelin or DsRed for controls (Fig. 4A). Previously, Hack et al. (2002) performed similar experiments and described a detachment effect of Reelin at 48 hours after plating, but no effect on chain extension. We decided to perform a dynamic analysis of cell migration from 12 to 72 hours in order to better characterize the effect of Reelin on SVZ-derived cell migratory behavior. After 12 hours in culture, chains start to emerge from the explants in both control and Reelin conditions, indicating that even in presence of Reelin, the initiation of migration occurs in chains (Figure S4A,B), with no obvious difference in chain length but subdued modifications in the structure of the chains that appear less compacted in presence of Reelin. Twenty four hours after plating, we clearly observed the detachment effect of Reelin [11], i. e. more cells escaping from chains of neuroblasts (Fig. 4B–F), which then progressed individually. We estimated the migration distance (distance between the edge of the explant and the migrating front of chains) proximal or distal to the HEK aggregate in the two groups. No significant difference was observed in migration orientation, thereby indicating that Reelin is not chemoattractant to SVZ progenitors (Fig. 4G). Interestingly, we also showed that the migration distance was significantly enhanced by 30% in the Reelin condition compared to the control (Fig. 4H). In order to further understand the migration response of SVZ cells to Reelin exposure, we analyzed the explants according to their distance to the Reelin source: the “close explants” located at a distance inferior to 500 µm and the “far explants” positioned more than 500 µm away from the source. Whatever the distance to the HEK-Rln aggregate, no chemoattraction could be detected (ratio chain length proximal vs distal was 1.03±0.1 in “close explants” and 1.02±0.15 in “far explants”). However, the detachment and the chemokinetic effects were still clearly observed in the most distant explants (mean chain length of 134.7±11.98 µm in HEK-DsRed vs 212.7±23.3 µm in HEK-Rln; p = 0.003), thus confirming the presence of Reelin at such distances from the source. These results suggest a threshold rather than a gradient effect of Reelin on SVZ cells.

At later time points (48 hours), chains begin to be quite disorganized in Reelin condition and cell detachment starts to occur even in control conditions. Because of this disorganization, chain length is no more significantly different at 48 hours between control explants and Reelin explants (Figure S4C–G), in accordance with previous studies reporting no chemokinetic effect of Reelin at 48 hours (Hack et al., 2002)

This is the first demonstration that Reelin does not only promote detachment but also chain extension in postnatal SVZ explants. To further complete this new and unexpected observation, we performed video time-lapse microscopy. Twenty four to 48 hours after explants plating, individual cells fully detached from the chains were traced over 9 hours (192 cells in control and 298 cells in Reelin condition, in 3 independent experiments) (Fig. 4I–K). The mean migration speed of isolated cells was significantly enhanced by 36% in the presence of Reelin in the culture medium (19.0±1.6 µm/h vs 14.4±2.1 in control condition, p = 0.04; Fig. 4 K), thereby revealing a chemokinetic effect. The tortuosity of cell trajectory was not influenced by the presence of Reelin (2.1±0.2 in control vs. 2.3±0.6; p = 0.73), in concordance with the absence of a guidance effect of Reelin described in the previous experiment.

On the basis of these results, we conclude that Reelin is not a directional cue; on the other hand, it does enhance chain detachment and NSPC migration speed in vitro both of cells migrating cooperatively (in chains) and individually.

Based on our in vivo observations (Fig. 3M,O), we also examined the effect of Reelin on neuronal maturation of SVZ-derived progenitors. In explant experiments, cells were labeled with calcein at different time points and neurite length was monitored. Up to 24 hours in culture, we did not observe any difference in the morphology of neuronal progenitors, nor in total neurite length, in control and Reelin conditions (Figure S5A). However, after 72 hours, cell morphology appeared more complex (not shown) and neurite length was increased in presence of Reelin (Figure S5A–C). To better characterize the effect of Reelin on differentiation, we performed dissociated SVZ cell culture on polylysined coverslips and Tuj1 immunolabeling (Figure S5D–G). The presence of Reelin did not influence the neuronal fate of SVZ-derived progenitors (77.3±1.9% vs 77.8±2.6% Tuj1+ cells in control and Reelin conditions respectively; p = 0.50), but again, we observed an effect of Reelin on neuronal maturation (enhanced neurite length and more complex ramification) after 72 hours of culture, whereas after 24 hours nor total neurite length nor arborization complexity was affected (Figure S5D–G). We then examined the direct effect of Reelin on SVZ-derived OPCs in vitro in order to check for a possible effect on oligodendrocyte maturation. Addition of Reelin into the culture medium did not significantly affect the engagement of SVZ progenitors into the oligodendrocyte fate nor their maturation assessed by the proportion of O4/Olig2 (p = 0.55) and MBP/Olig2 (p = 0.10) labeled cells (Figure S6).

These results indicate that Reelin can have differential effects on SVZ derived progenitors depending on the length of exposure, acting on migration speed, detachment and differentiation sequentially.

### Reelin overexpression favors the recruitment of endogenous NSPCs in a model of demyelination

**Figure 5:**
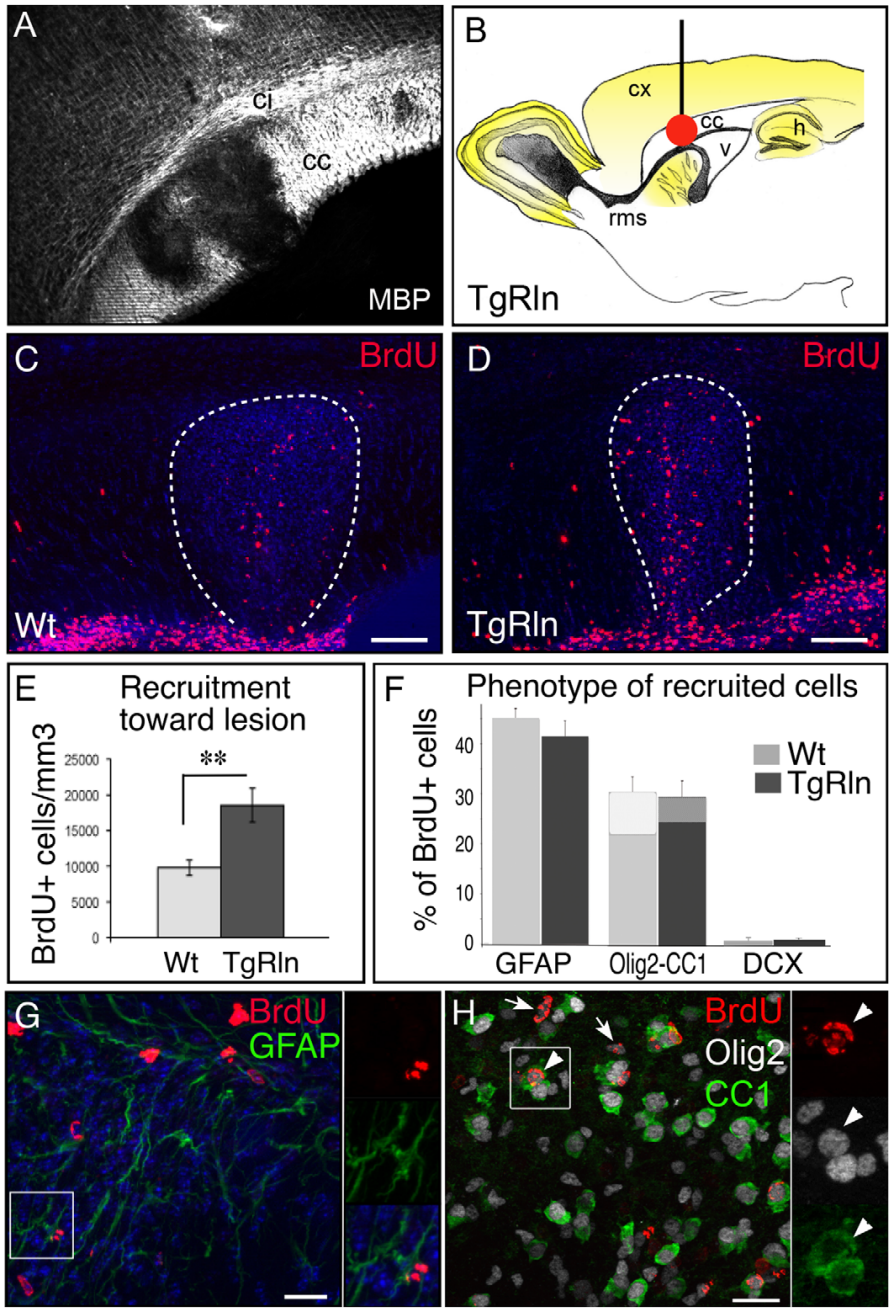
Mobilization of SVZ-derived NSPCs toward a lysolecithin-induced focal demyelination lesion in the corpus callosum is increased in transgenic mice overexpressing Reelin and inhibited in absence of Reelin signaling. A: Illustration of lysolecithin-induced demyelination in the corpus callosum. The demyelination lesion is revealed by myelin basic protein (MBP) immunostaining. B: Schematic representation of demyelination lesion induction in the corpus callosum of TgRln mice overexpressing Reelin (yellow) in forebrain structures. C, D: BrdU labeling evidencing SVZ-derived NSPCs migrating in the RMS and recruited toward the demyelination lesion delineated by white doted line, in Wt (C) and TgRln (D) mice. E: Quantification of BrdU+ SVZ-derived cells within the lesion site showing a highly significant increase in cell recruitment in transgenic mice overexpressing Reelin as compared to their Wt littermates. F: Phenotype of SVZ-derived progenitors recruited at the lesion site 7 days after LPC injection. Recruited cells mostly give rise to astrocytes (GFAP-positive) and to cells of the oligodendrocyte lineage (Olig2-positive). Among BrdU/Olig2 labeled cells, a subpopulation also expresses CC1, a marker of mature oligodendrocytes (represented by lighter gray at the upper part of the columns). Only a negligible proportion of recruited cells adopt neuronal fate. Reelin overexpression does not seem to have major impact on the fate of recruited cells. G: Double labeling for BrdU (red) and GFAP (green) in the lesion site. H: Triple labeling for BrdU (red), Olig2 (white) and CC1 (green) in the lesion site. Arrows indicate BrdU-Olig2 co-labeled cells; arrowhead indicates one BrdU-positive cell also labeled with Olig2 and CC1. Small white squares (in G and H) are enlarged at the right side of the picture to show the co-labeling. TgRln: transgenic mice overexpressing Reelin; Wt: Wt mice. Scale bars  = 100 µm.

To assess the extent to which Reelin-induced NSPC dispersion and migration is functionally beneficial to enhance spontaneous repair processes, we induced a focal demyelination lesion by lysolecithin injection in the corpus callosum of Wt and TgRln animals (Fig. 5A,B). Seven days later, the lesion was clearly visible around the injection point in the corpus callosum by the loss of myelin basic protein (MBP) labeling (Fig. 5A). The lesion size was similar in both groups (0.31±0.05 mm^3^ in Wt mice and 0.25±0.09 mm^3^ in TgRln mice; ns). SVZ-derived cells were traced using BrdU injections one day before inducing the lesion (see methods). We found a strong and significant (p<0.01, n = 5) increase in recruitment, with twice as many BrdU+ cells within the lesions in TgRln mice compared to Wt littermates (Fig. 5 C–E). We did not measure any difference in SVZ/RMS cell proliferation or survival that could account for this variation (see Pujadas et al., 2010 in healthy brain, and see Figure S7A,C in supplementary material for lesioned brain). Interestingly, the ratio of SVZ-derived cell density inside vs outside the lesion (i.e. in the rest of the ipsilateral CC) was more than doubled in TgRln mice (8.3±0.8 in Wt vs 17.3±3.5 in TgRln, p = 0.01). This observation supports the notion that once the cells had escaped the SVZ/RMS they migrated preferentially toward the lesion.

We then examined the phenotype of SVZ-derived recruited cells at the lesion site, 7 days after LPC injection (Fig. 5F–H). Both in Wt and TgRln animals, around 40% of the BrdU+ cells in the lesion gave rise to GFAP+ astrocytes (45.4±1.9% and 41.6±3.1% in the Wt and TgRln respectively; n = 5, p = 0.21), another 30% became committed to the oligodendrocytic lineage (30.8±3.1% and 29.8±3.2% of Olig2+ cells in the Wt and TgRln respectively; n = 5, p = 0.5). Olig2 is a pan-oligodendroglial cell marker, from early progenitors to myelinating oligodendrocytes. In order to evaluate oligodendrocyte maturation, we performed triple immunolabeling (BrdU/Olig2/CC1) since CC1 is expressed only by mature oligodendrocytes. As previously observed at this stage [15], many of the Olig2-positive cells were still immature since only a minority of them expressed CC1, with a slight reduction of maturation in TgRln mice (28.2±3.2% and 18.1±2.5% of Brdu/ Olig2-positive cells were also CC1-positive in Wt and TgRln respectively; p = 0.03). Finally, less than 1.2% of recruited cells showed a neuronal fate in both groups (DCX+). In summary, Reelin did not influence the cell fate of recruited NSPCs.

In conclusion, by modifying progenitor migratory behavior, Reelin overexpression led to increased cell recruitment at the lesion site.

### Impaired Reelin signaling strongly inhibits spontaneous NSPC recruitment in a model of demyelination

Finally, in order to examine the role of Reelin expression in spontaneous endogenous NSPC recruitment, we disrupted Reelin signaling either genetically (using scrambler mice that are knock out for Dab1, the intracellular adaptor required for Reelin signaling), or using a Reelin blocking antibody.

**Figure 6:**
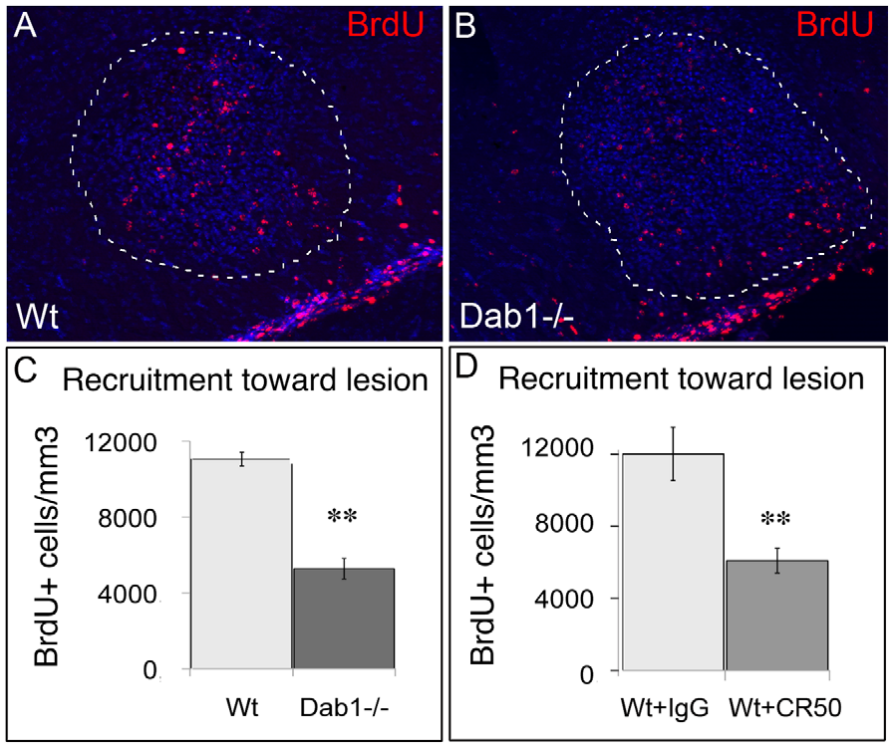
Lysolecithin-induced endogenous progenitor cell recruitment is strongly reduced in mice lacking Reelin signaling. A, B: BrdU labeling evidencing SVZ-derived NSPCs migrating in the RMS and recruited toward the demyelination lesion delineated by white doted line, in wild-type (C) and Dab1−/− mice (D). C, D: Quantification of BrdU-positive SVZ-derived cells within the lesion site showing a significant decrease in cell recruitment in mice lacking the Dab1 adaptor required for Reelin signaling (C) as well as in Wt mice treated with Reelin blocking antibody CR50 (D). Scale bars: 50 µm.

We observed a strong and significant reduction (52%; p = 0.01, n = 4) in the number of SVZ-derived cells at the lesion site in *Dab1^−/−^* mice compared to Wt littermates (Fig. 6A–C). We checked that no difference in proximal RMS size (Fig. 6A,B) and no significant alteration of SVZ cell proliferation or survival (Figure S7B,D) could account for this reduction. Since F-spondin (Spon1) has been shown to regulate chain migration in the RMS of postnatal mice via Reelin receptors and Dab1 signaling [16], we analyzed Spon1 expression in healthy and lesioned mice. Spon1 was still detected in the SVZ/RMS of adult Wt as well as TgRln mice (Figure S8A), but we did not observe any upregulation of this protein after lesion, either in the SVZ/RMS or in peri-lesional structures (Figure S8B,C).

In order to reinforce these results, we also injected blocking Reelin antibody (CR50) after LPC lesion in Wt mice. We quantified twice less recruited cells at the lesion site in mice that received CR50 antibody as compared to control mice injected with non specific IgG (Fig. 6D).

We conclude that spontaneous post-lesional NSPC mobilization is impaired in the absence of Reelin signaling.

## Discussion

In the present study, we demonstrate that Reelin, a developmental migration cue, continues to play a crucial role in progenitor cell migration in the postnatal brain. Although largely down-regulated after the end of development, we show that Reelin is reactivated under pathological conditions in the adult brain. This re-expression contributes to spontaneous endogenous NSPC recruitment at the lesion site and can be experimentally forced for increased cell replacement. This augmented recruitment is not due to chemoattraction but results from Reelin's action on the velocity, chain detachment and dispersion of NSPCs during migration that we observed in vitro and/or in vivo.

### How does Reelin influence cell migration?

During cortical development, Reelin, acts as a positional cue by mechanisms that remain to be elucidated, although recent studies have uncovered its role on cytoskeleton stabilization through cofilin phosphorylation [17] and on neuronal polarization via extension of the Golgi apparatus into the apical dendrites [18]. Two models have been put forward: the “detach and stop” and “detach and go” models, in which Reelin either induces the detachment of neurons from radial glia and then triggers their arrest and differentiation, or stimulates somal translocation respectively [19]. In the adult brain, Reelin allows cell dispersion in the OB via a detachment effect on neuroblasts migrating in chains [11] through Dab1 phosphorylation and activation of the ERK pathway [20]. A recent study using Reelin knockouts challenged this view by showing that chains do not form from SVZ-derived neurospheres and cells migrate out of the neurospheres in a dispersed way [21]. Our in vivo experiments showed that ectopic Reelin expression in the adult brain triggers cell detachment and recruitment from the SVZ/RMS toward the corpus callosum regardless of the location of the Reelin source, thereby suggesting the absence of a chemoattraction effect. This notion is in accordance with our in vitro experiments, in which migration from SVZ explants was regularly distributed over all sides of the explants independently of the position and the distance of Reelin source. In addition, we also clearly observed an unsuspected chemokinetic effect both on chains and on neuroblasts migrating individually. In vivo, we also noticed that Reelin induced cells to leave the RMS either individually or in chains.

Altogether, our in vivo and in vitro observations suggest that in the adult brain, Reelin acts as a positioning cue following the “detach and go” scenario.

Interestingly, neural progenitors were also found to exhibit a more complex and ramified morphology when exposed to Reelin, both in vivo and in vitro. These observations are consistent with studies reporting that Reelin promotes neuronal differentiation and dendritic development [21], [22], [23], [24]. This dual effect of Reelin both on migration and differentiation may appear paradoxical. However our dynamic in vitro analysis suggests that, depending on the duration of exposure, Reelin has sequential effects on SVZ-derived progenitors, first by triggering detachment and migration, second by promoting differentiation.

Since Reelin favors neuronal differentiation, it could be of great interest to address in further studies its potential benefits in other neurodegenerative models.

### Reelin, a key player of plasticity after brain lesion?

We showed that Reelin reactivation was conserved in two brain lesion models involving distinct mechanisms (ischemia or chemical toxicity) and different cell types (global cell loss or specific oligodendrocyte loss). Thus, it appears to be a general response of the brain to different types of damage. Reelin up-regulation after lesion was previously reported in several tissues other than the brain, such as the liver [25], peripheric nerves [26] and ocular tissue [27]. This conserved and ubiquitous implication of the Reelin pathway in lesions of various tissues supports the notion that this protein exerts a crucial role in the regulation of cell plasticity in pathological processes.

In physiological conditions, Reelin is weakly expressed in the adult cerebral cortex, this expression being mainly in GABAergic interneurons [28]. The functional significance of this postnatal Reelin expression in cortical GABAergic interneurons remains poorly understood [29]. After lesions, Reelin expression in cortical interneurons is increased and a greater number of them are Reelin+. Therefore, some interneurons appear to have the capacity to re-activate Reelin expression after lesion in the adult brain. Spontaneous Reelin up-regulation remains relatively moderate compared to the 3–6-fold increase in Reelin levels observed in TgRln mice, accounting for enhanced endogenous cell recruitment in this animal model. We observed that the proportion of cells that reached the lesion among those that exited the SVZ/RMS was greatly and significantly enhanced in TgRln . Whereas Reelin does not show any chemoattractant effect, brain lesions are associated with secretion of a panel of cytokines such as MCP-1 and TNF-alpha [30], [31] which are themselves chemoattractant. Due to its detachment effect, Reelin potentiates the chemoattractant effect of lesion-induced cytokines. Indeed, once they have escaped the rostral migratory stream and reached the corpus callosum, SVZ-derived progenitors will preferentially migrate toward the lesion site. These results support our hypothesis that the exit from SVZ/RMS pathway is a key step for efficient progenitor recruitment to the lesion.

In addition, our experiments in *Dab1^−/−^* mice and in presence of Reelin blocking antibody showing reduced NSPC recruitment at the lesion site confirm that Reelin signaling participates in spontaneous cell mobilization. Our data shed new light on a previous study reporting increased susceptibility of Reelin-deficient mice to ichemic brain injury [32]. Altogether these results led us to propose that the endogenous up-regulation of Reelin after a lesion facilitates the exit of SVZ-derived progenitors from the RMS and thereby promotes their recruitment toward a demyelination lesion in the adult brain.

These results nicely show how a key developmental cue is reused to control the regenerative processes in the adult brain.

### Conclusion

In conclusion, our study reveals a new role for Reelin in the healthy and in the injured adult brain and provides the proof of concept that targeting endogenous SVZ/RMS cell migration is a valuable strategy to promote regenerative processes. In humans, SVZ as a niche of neural stem cells is now largely admitted, and post-lesional reactivity as well as migratory potential have been reported [33], [34], [35]. However, the presence of a RMS is still a matter of debate [36], [37], [38], [39] although increasing number of studies suggests the existence of an SVZ extension that may be assimilated to a RMS [34], [37], [40]. Therefore, promoting brain repair by mobilizing endogenous pools of NSPCs may also be possible in humans.

Recent studies have demonstrated that the presence of NSPCs at the lesion site is beneficial for brain repair by mechanisms other than cell replacement, notably by anti-inflammatory, neuroprotective and neurotrophic effects [41], [42]. Developing strategies to enhance endogenous NSPC recruitment thus presents a double advantage: first, progenitors that complete differentiation could replace lost cells and those that remain immature could contribute to immunomodulation and neuroprotection.

## Materials and Methods

### Cell cultures

Unless specified, all culture reagents were purchased from Gibco.

#### HEK cell line

Reelin (pCrl, kind gift from A.M. Goffinet [43], and DsRed constructs (pDsRed2-N1, Clontech) were stably expressed in HEK 293 cells (gift from R. Seidenfaden) maintained in DMEM, 10% fetal bovine serum, 1% Na-Pyruvate MEM, and 1% penicillin/streptomycin. HEK cell aggregates were generated using the hanging drop method.

#### Primary cultures of SVZ explants

SVZ explants were obtained as previously described [44] from P1–P3 newborn mice. Explants were mixed with Matrigel in a 1∶2 ratio (BD Bioscience) and disposed equally distant to a central HEK cell aggregate expressing Reelin (HEK-Rln) or DsRed (HEK-DsRed) in defined medium (DMEM:F12 (3∶1), insulin (5 µg/ml), Apo-transferin (100 µg/ml), putrescine (100 µM), progesterone (20 nM), selenium (30 nM) and penicillin/streptomycin (100 UI/ml / 100 µg/ml) supplemented with 2% B-27.

Explants were analyzed (Colibri microscope, Zeiss) 12, 24, 48 and 72 hours after plating. The number of cells detached from the chains was quantified at 24 hours, and the length of migratory chains was determined at 24 and 48 hours (in proximal and distal positions relative to the HEK aggregate in 3 independent measure points, 10° apart, see Fig. 4A).

For time-lapse experiments, SVZ explants were placed on Matrigel coating (1/50) in the center of the dish, and the HEK cells (HEK-Rln or HEK-DsRed for controls) were seeded around the dish periphery. Recording started 24 to 48 h after plating for 9 h (6 image/h, x10 magnification, thermostated inverted Colibri microscope (Zeiss, 37°C, 5% CO2)). To limit bias of cell-cell interaction, only SVZ progenitors at the periphery of the explants that had no contact with other cells were traced. Cell trajectories, mean migration speed and tortuosity (ratio of the total length of the curve on the straight distance between the 2 ends) were calculated using Axiovision software (Zeiss).

For neuronal maturation analysis, explants were incubated with calcein (4 µg/ml 1 h at 37°C), and neurite length was measured using Axiovision software (Zeiss).

#### Primary cultures of dissociated SVZ cells for differentiation studies

Since 3D matrigel cultures were not the best conditions to study neuronal differentiation, we also used dissociated SVZ cells cultured on poly L-lysine (Sigma) coated coverslips to perform morphometric analysis. After 1 or 3 days in culture in conditioned media from HEK cells, with 0,1% fetal calf serum but without growth factors, cells were fixed (paraformaldehyde 4%, 10 minutes) and neurons were labeled using anti-Tuj1 antibody (Covance, 1/1000) to visualize neuritic branches. Neurite length and total number of branches per neuron were measured using Axiovision software (Zeiss). Cells of the oligodendrocyte lineage were labeled with mouse IgM anti-O4 antibody (1/2; our hybridoma production), rabbit anti-Olig2 (1/1000, Chemicon) and rat anti-MBP (1/100, Chemicon) for myelin.

#### Primary cultures of SVZ NSPCs grown as neurospheres and nucleofection

Neurospheres were produced as previously described [45] from P3–P5 actin-GFP newborn mice, in defined media supplemented with 2% B27 and 20 ng/ml EGF and bFGF (R&D System). 24 h after the 3d passaging, neurospheres were nucleofected with pCrl or DsRed plasmids using Amaxa Biosystems, following the manufacturer's instructions.

### Biochemistry

#### Sample preparation

3 days after thermocoagulation or demyelination lesion, structures surrounding the lesion and their counterparts in the contralateral hemisphere were dissected on 1 mm coronal sections in cold HBSS (cortex for thermocoagulation, and corpus callosum together with adjacent parts of cortex and striatum for LPC induced demyelination). Tissues were freshly crushed and sonicated in homogenizing buffer (100 mg tissue/ml in 50 mM Tris-HCl pH 7.5, 150 mM NaCl, 1 mM EDTA, 1 mM PMSF and anti-proteases) and supernatant collected after centrifugation (1 h, 10 000 g, 4°C).

#### Western Blotting

Supernatant proteins (60 µg load, quantified by Bradford assay) were resolved by SDS/PAGE on 4%–12% gels (Invitrogen) and blotted onto PVDF membranes. For Western blotting, membranes were blocked for 1 h in TBS-T (0.02 M Tris pH 8, 0.5 M NaCl, 0.1% Tween-20) containing 4% dehydrated half-creamed milk, and incubated (ON, 4°C) with mouse anti-Reelin (1/2 000, Chemicon), rabbit anti-F-spondin (R1, 1/3000 kind gift from Dr A. Klar) or mouse anti-a-tubulin (1/30 000, Sigma) then with anti-mouse horseradish peroxidase-conjugated antibodies (1∶3 000; 2 h RT; Jackson ImmunoResearch) and ECL substrate (Lumi-Light Western Blotting Substrate; Pierce chemical company). Chemiluminesce was registered under ECL Imager Chemi-Smart system (Fisher Bioblock). Semi-quantitative analysis of Reelin expression was performed using Bio-1D advanced Software (Vilmer Lourmat), with a-tubulin as an internal control.

### Animals

All experimental and surgical protocols were performed following the guidelines established by the French Ministry of Agriculture (Animal Rights Division). The architecture and functioning rules of our animal house, as well as our experimental procedures have been approved by the "Direction Départementale des Services Vétérinaires" (ID number E-13-055-21).

Surgeries and perfusions were done under anesthesia with an intraperitoneal injection of xylazin (10 mg/kg, Bayer) and ketamin (100 mg/kg, Merial); body temperature was kept close to 38°C.

We used 2 month-old CD1 or nude males (Charles River) to prevent immune rejection of HEK cells. Conditional transgenic mice overexpressing Reelin (TgRln) were generated on a C57Bl6 background. Reelin expression is driven in postnatal forebrain neurons by tet-off system under the control of the pCamKIIα promotor (see Pujadas et al., 2010 for details and doxycyclin treatment). Scrambler mice (*Dab1^scm^*, The Jackson Laboratory; Sheldon et al., 1997) were bred in our animal facility on C57Bl6 background. Actin-GFP mice [46] were used as donor mice for SVZ cell grafting experiments in wild-type (Wt) or TgRln recipient mice.

#### Lesions

Focal demyelination by toxic insult using lysolecithin (LPC) intracerebral injection is a commonly used model of demyelination in rodents resulting in local specific oligodendrocyte cell death and moderate inflammation. In parallel, we also used a model of cortical ischemia induced by thermocoagulation of meninges, which leads to extended cortical neurodegeneration and inflammation [47].

#### Focal demyelination

LPC focal demyelination was performed as described by [48] with minor modifications. Briefly, either 2-month-old CD1, 2-month-old TgRln mice and Wt littermates, or 2–4-month-old scrambler (Dab1−/−) and age matched C57bl6 Wt mice, were anesthetized and placed in a stereotactic frame (Kopf). 0.7 µl of a solution of 1% LPC (Sigma) in 0.9% NaCl was injected unilaterally into the corpus callosum (1.5 mm anterior, 1 mm lateral to Bregma, 2 mm deep from cortical surface). Animals were killed 3 or 7 days later, a delay adequate to observe the demyelinated lesion before spontaneous remyelination occurs.

For blocking experiments, 2 µl of undiluted Reelin blocking antibody (CR50, MBL, 1 µg/µl) were injected at the same site consecutively to LPC injection.

#### Thermocoagulation

Unilateral ischemic lesions were induced by thermocoagulation of pial blood vessels, as described by [49]. Briefly, a window was opened in the skull overlying the right frontoparietal cortex and pial arterioles were coagulated with a heated probe. Animals were killed 3 or 7 days later.

#### NSCP tracing

The thymidine analog BrdU was used to trace SVZ-derived cells as described in [50]. One day before surgery, the animals received 4 BrdU injections (100 mg/kg i.p., Sigma) at 2 h intervals. Using this protocol, cells integrating BrdU at the time of injection (i.e. before any lesion had been performed) are restricted to the SVZ and RMS.

Repeated injections with these doses of BrdU allowed us to strongly label a large proportion of the NSPC population without reaching toxic doses and without staining of dying cells [50], [51].

Therefore, 7 days after lesion, labeled cells detected in periventricular structures and lesion areas are assumed to originate from the SVZ or the RMS [52].

#### Grafts

Mice were anesthetized, placed in a stereotactic frame (Kopf), and nucleofected neurospheres (10,000 cells in 0.6 µL HBSS) or stably transfected HEK cells (50,000 cells in 0.5 µL HBSS) were injected into subcortical white matter tracts (+1 mm anterior to Bregma, −1 mm lateral, and −2 mm deep from cortex surface) as described in [53], or into right lateral ventricle (+0.2 mm anterior to Bregma, −1 mm lateral, and −2.2 mm deep from cortex surface) respectively. Animals were killed 7 days after grafting.

To analyze individual cell migration within the RMS, freshly dissociated SVZ cells from actin-GFP mice (20,000 cells in 1 µL HBSS) were injected into the RMS of adult TgRln and Wt mice (+1.4 mm anterior to Bregma, −1 mm lateral, and −2.3 mm deep from cortex surface). Animals were killed 7 to 10 days after grafting.

### Histology

#### Immunohistochemistry

After intracardiac perfusion with 4% paraformaldehyde, brains were removed, postfixed for 2 h, and cut on vibratome (Leica) in 4 series of sagittal or coronal sections (50 µm).

We used the following primary antibodies: mouse IgG1 anti-Reelin (1/200, Chemicon), rabbit anti-GFP (1/200, Invitrogen), rat anti-BrdU (1/500, AbCys), mouse IgG1 anti-MBP (1/500, Euromedex) for myelin, rabbit anti-Olig2 (1/500, Chemicon) and mouse IgG2 anti-CC1 (1/500, Calbiochem) for oligodendrocyte lineage, mouse IgG1 anti-GFAP (1/500, Sigma) for astrocytes, rat anti-CD3 (1/100, Serotec) for T cells, and mouse anti-ßIII tubulin (1/1000, Eurogentec) or goat anti-Doublecortin (DCX, 1/250, Santa Cruz) for migrating neuronal precursors, rabbit anti-phospho-histone 3 (1/250, Upstate) for mitotic cells, rabbit ant-caspase3 (1/200, Cell Signaling) for apoptotic cells and rabbit anti-GABA for interneurons. Isolectin B4 staining (IB4) (1/100, Sigma) was used to identify microglial cells and macrophages. Secondary antibodies coupled to FITC, Texas Red, Cy3 or Cy5 (1/200 in PBST−0.1%, Interchim Jackson) or Alexa488 or Alexa555 (1/500, Invitrogen Molecular Probes) were used. For BrdU immunocytochemistry, sections were first incubated for 20 minutes in 2N HCl in PBS containing 0.3% Triton X100 at 37°C, and then for 5 minutes in 0.1 M borate buffer (pH 8.5). For Reelin/NeuN double labeling (both mouse IgG1 antibodies), we used the Zenon kit, following the manufacturer instructions (Molecular Probes). Negative controls were performed by omitting primary antibodies. Sections were then counterstained with Hoechst 33342 (1/1000, Sigma).

#### In situ hybridization

In situ hybridization was performed as previously described in [11]. Briefly, antisense digoxigenin (DIG)-labeled riboprobe for Reelin was produced from a cDNA pBluescript plasmid (gift from A. Goffinet). Freshly cut vibratome sections were post-fixed for 10 minutes with 4% paraformaldehyde, treated for 5 minutes at room temperature with 1 µg/ml Proteinase K (Roche) in 10-mM Tris (pH 7.5). Hybridization was performed with 400 ng/ml of anti-sense riboprobe (or sense for controls) and revelation was done with anti-DIG-alkaline-phosphatase-conjugated antibody followed by NBT/BCIP staining.

#### Analysis and quantification

Cells were counted in one every other four sections through the whole structure. The area of the structures of interest was measured using the Explora Nova software. The number of BrdU+ cells within the demyelinated lesion was counted using a semi-automated method, as described in [50]. Colocalization of cell-specific markers was determined on multi-labeled sections at 40X magnification using confocal imaging with the Apotome system (Zeiss). For quantification of Reelin signal intensity, densitometry of single Reelin+ cells was measured using axiovision (Zeiss) software.

### Statistical analysis

The values presented are means ± s.e.m. For independent two-group comparisons, the data were statistically processed with the non-parametric Mann-Whitney test. For paired sample analysis, the Wilcoxon test was used. When more than two groups were compared, an ANOVA was performed before post-hoc analysis. P≤0.05 was considered significant and p≤0.01 highly significant. All measurements and subsequent evaluation were performed blind to the experimental group to which the animals belonged.

## Supporting Information

Figure S1 **Cells reactivating Reelin expression in peri-lesional areas are not astrocytes (GFAP-negative, A**–**C), nor oligodendrocytes (Olig2-negative, D**–**F), nor infiltrated blood cells (CD3-negative, G**–**I), nor microglia (IΒ4-negative, J**–**L) in the ipsilateral cortex 3 days after lesion.** Scale bars: A–I = 20 µm, J–L = 10 µm.
(TIFF)

Figure S2 **Transiently nucleofected neurospheres correctly express Reelin** ***in vitro*** **and** ***in vivo*** **.** A–C: Immunolabeling of GFP (green, A, C) and Reelin (red, B, C) showing that neurospheres nucleofected with pCrl plasmid still express Reelin 7 days after grafting in the cingulum. D: Western blot analysis of culture supernatant 24 hours after nucleofection of DsRed (DsR) or pCrl (Rln) plasmid and of stable HEK cell line overexpressing Reelin (HEK). Scale bars: A–C = 10 µm.
(TIFF)

Figure S3 **In TgRln mice, cells that leave the RMS migrate either individually or in chains, indifferently toward the striatum or the corpus callosum, and exhibit complex morphology.** A–B: Immunolabeling of GFP (grafted SVZ cells, in green) and DCX (endogenous neuroblasts, in red) in TgRln mice, showing that GFP+ grafted cells that escape from the RMS can orientate either toward the corpus callosum (cc) or toward the striatum (St). Some of them migrate individually by detaching from other progenitors (arrow in A) while others escape following DCX positive chains that are derived from the stream (arrowhead in B). Note the particularly mature morphology of detached grafted cells (arrow in A). C–E: Illustration of a grafted cell (GFP-positive) that escape the RMS in a TgRln mouse, and loose ßIII tubulin expression (in red). Scale bars  = 10 µm.
(TIFF)

Figure S4 **Dynamic analysis of cell migration from SVZ explants in absence and presence of Reelin.** Illustrations at 12 hours (A), 24 hours (B) and 48 hours (C) after plating. Note that pictures in C–D and E–F show the same explants at 24 and 48 hours. A,B: Effect of Reelin on early steps of cell migration from SVZ explants in vitro. The first signs of cell migration can be observed 12 hours after plating. Even in presence of Reelin in the culture medium, cells emerge from the explants performing chain migration. No obvious difference in chain length can be observed at that time between the two conditions. However, chains appear to be slightly less compacted in presence of Reelin (see magnification in boxes). C, D: 24 hours after plating, both cell detachment and chain length are notably increased in presence of Reelin. E, F: At longer time point (48 hours), the effect of Reelin on chain length is no longer significant. Cell detachment from the chains is becoming visible in control condition but it is still more obvious in presence of Reelin. G: Quantification of migration distance at 24 and 48 hours in absence and presence of Reelin. Scale bar: A, B = 50µm; C–F = 100µm.
(TIFF)

Figure S5 **Effect of Reelin on SVZ-derived neuronal progenitor maturation** ***in vitro*** **.** A–F: effect of Reelin on total neurite length, either in pro-migratory conditions (explant cultures (A–C)) or in differentiation conditions (dissociated cell culture (D–F)) 24 and 72 hours after plating. Neurite length is not affected at 24 hours but is increased in presence of Reelin after 72 hours. B,C and E,F: Illustration of typical morphology of SVZ-derived progenitors in absence (B,E) or presence (C,F) of Reelin, after 72 hours of culture. G: Complexity of neuritic arborisation of SVZ-derived Tuj1-positive progenitors after 24 hours and 72 hours of culture. The presence of Reelin in the culture medium increases the number of branches per neuron at 72 hours but not at 24 hours. Scale bar: B–C = 20µm; E–F = 20 µm.
(TIFF)

Figure S6 **Effect of Reelin on SVZ-derived oligodendrocyte progenitor maturation** ***in vitro*** **.** A: Triple Olig2/O4/MBP immunolabeling of SVZ-derived neurospheres after 3 days differentiation in vitro, in presence (Rln) or absence (Ctl) of Reelin. B: quantification shows no effect of Reelin on the oligodendroglial fate or on the maturation of the progenitors.
(TIFF)

Figure S7 **SVZ cell proliferation and survival in TgRln and Dab1−/− mice with LPC lesion in the corpus callosum as compared to their wild type littermates.** No significant difference could be detected in the number of PH 3 or casp3-positive cells between wild type and transgenic mice. The number of mice analyzed is indicated inside each column, and the significance (Mann Whitney) indicated above each graph.
(TIFF)

Figure S8 **F-spondin expression in healthy and demyelinated (LPC) wild-type and transgenic mice overexpressing Reelin.** A: illustration of a western blot showing F-spondin expression in physiological conditions, in the SVZ/RMS of neonates (pool of 6 ten day-old mice) and adult mice (2 transgenic and 2 wild type mice). Recombinant human F-spondin (Spon1) was used as a positive control. F-spondin expression is low in adults but similar in Wt and TgRln mice. B–C: after LPC lesion, F-spondin expression is not significantly increased in SVZ/RMS (B) nor in perilesional structures (C) in Wt (n = 2) and TgRln mice (n = 3). F-spondin expression has been normalized to α-tubulin and expressed in percent of unlesioned side (contralateral).
(TIFF)
