# Supplementary figures and images for: Reelin Controls Progenitor Cell Migration in the Healthy and Pathological Adult Mouse Brain
Source: PLoS One. 2011 May 27;6(5):e20430. doi: 10.1371/journal.pone.0020430 (PMC3103550; doi:10.1371/journal.pone.0020430)

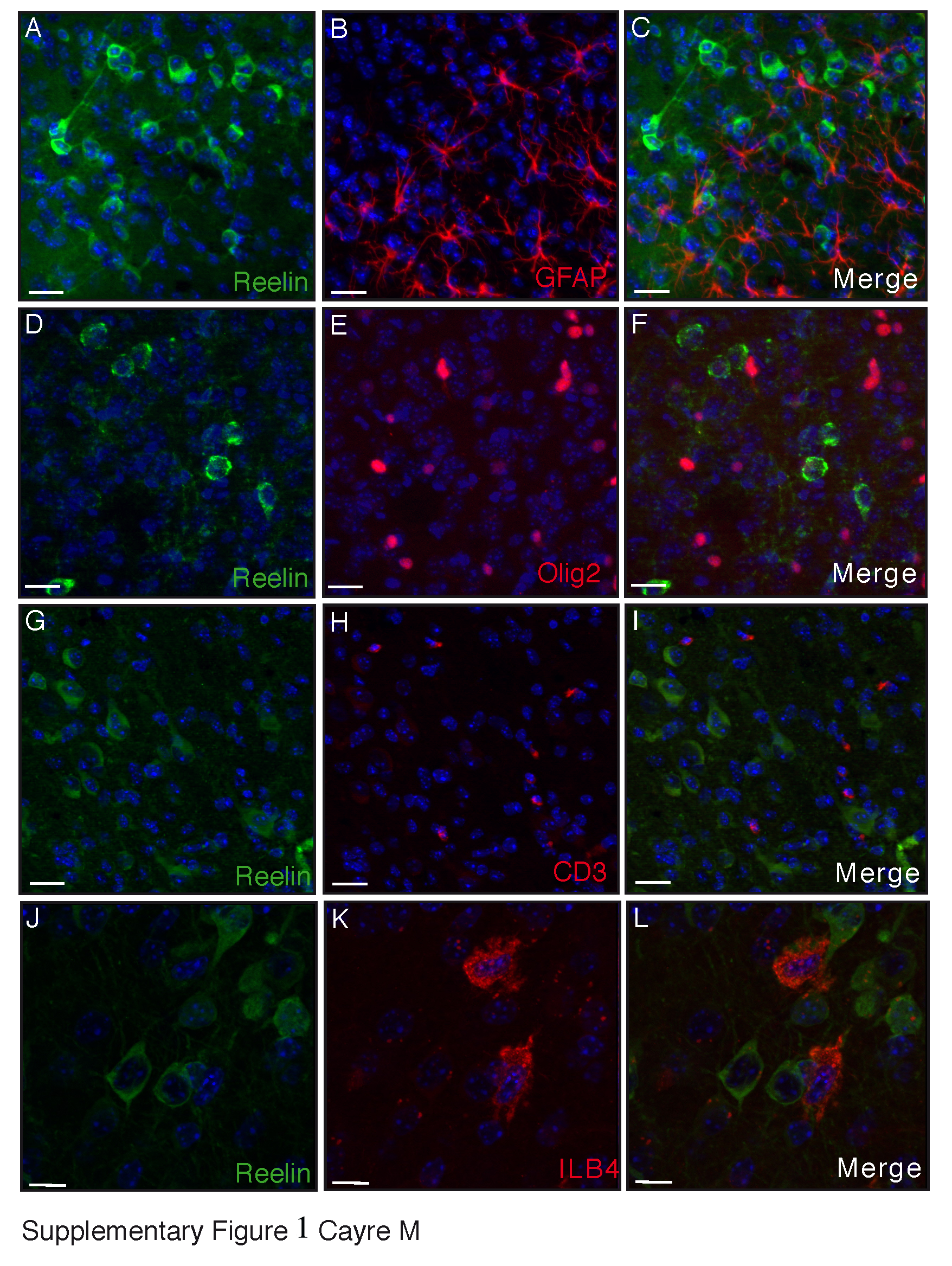

Supplement: Figure S1 — Cells reactivating Reelin expression in peri-lesional areas are not astrocytes (GFAP-negative, A–C), nor oligodendrocytes (Olig2-negative, D–F), nor infiltrated blood cells (CD3-negative, G–I), nor microglia (IΒ4-negative, J–L) in the ipsilateral cortex 3 days after lesion. Scale bars: A–I = 20 µm, J–L = 10 µm. (TIFF) [file pone.0020430.s001.tif]

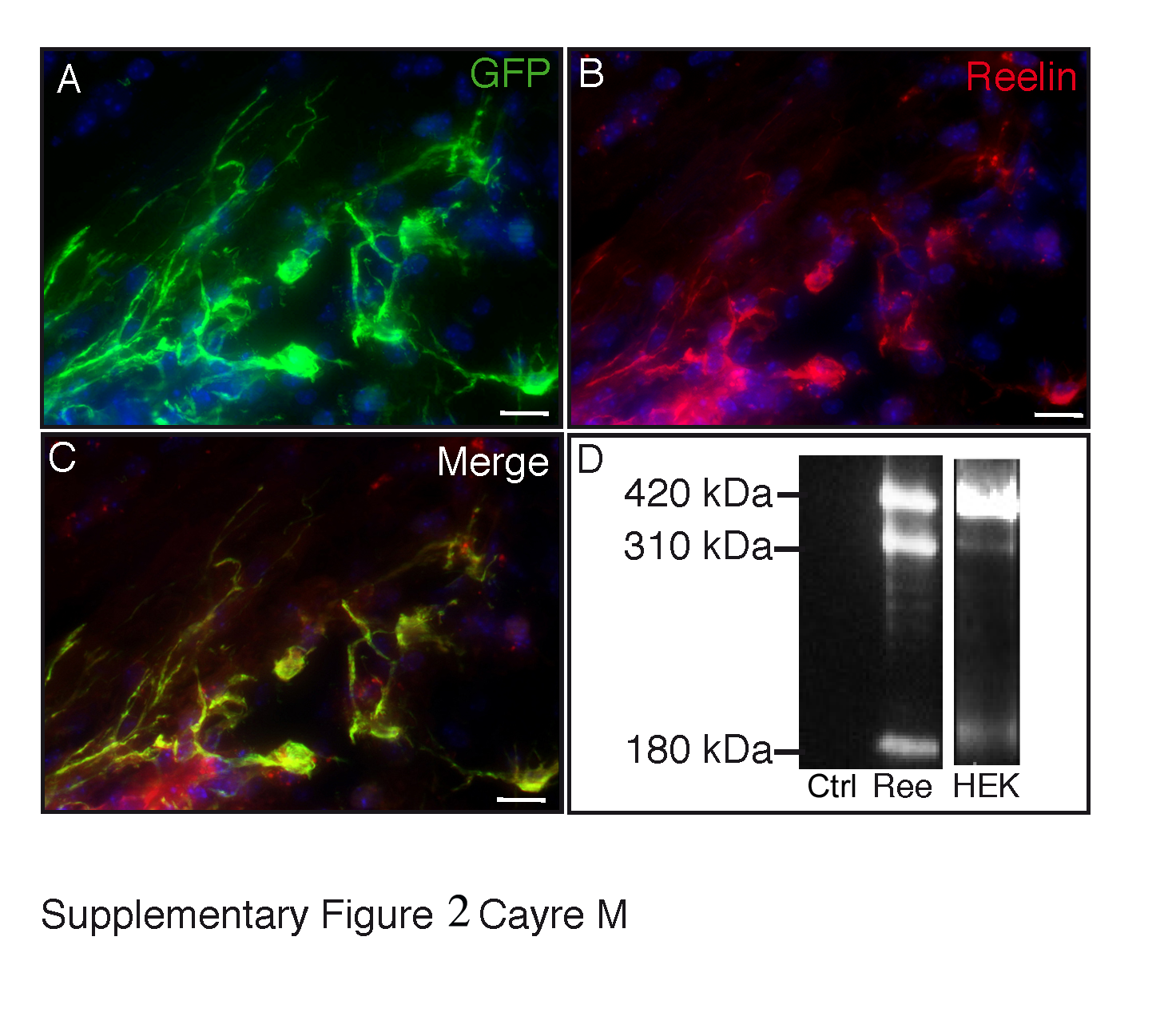

Supplement: Figure S2 — Transiently nucleofected neurospheres correctly express Reelin in vitro and in vivo . A–C: Immunolabeling of GFP (green, A, C) and Reelin (red, B, C) showing that neurospheres nucleofected with pCrl plasmid still express Reelin 7 days after grafting in the cingulum. D: Western blot analysis of culture supernatant 24 hours after nucleofection of DsRed (DsR) or pCrl (Rln) plasmid and of stable HEK cell line overexpressing Reelin (HEK). Scale bars: A–C = 10 µm. (TIFF) [file pone.0020430.s002.tif]

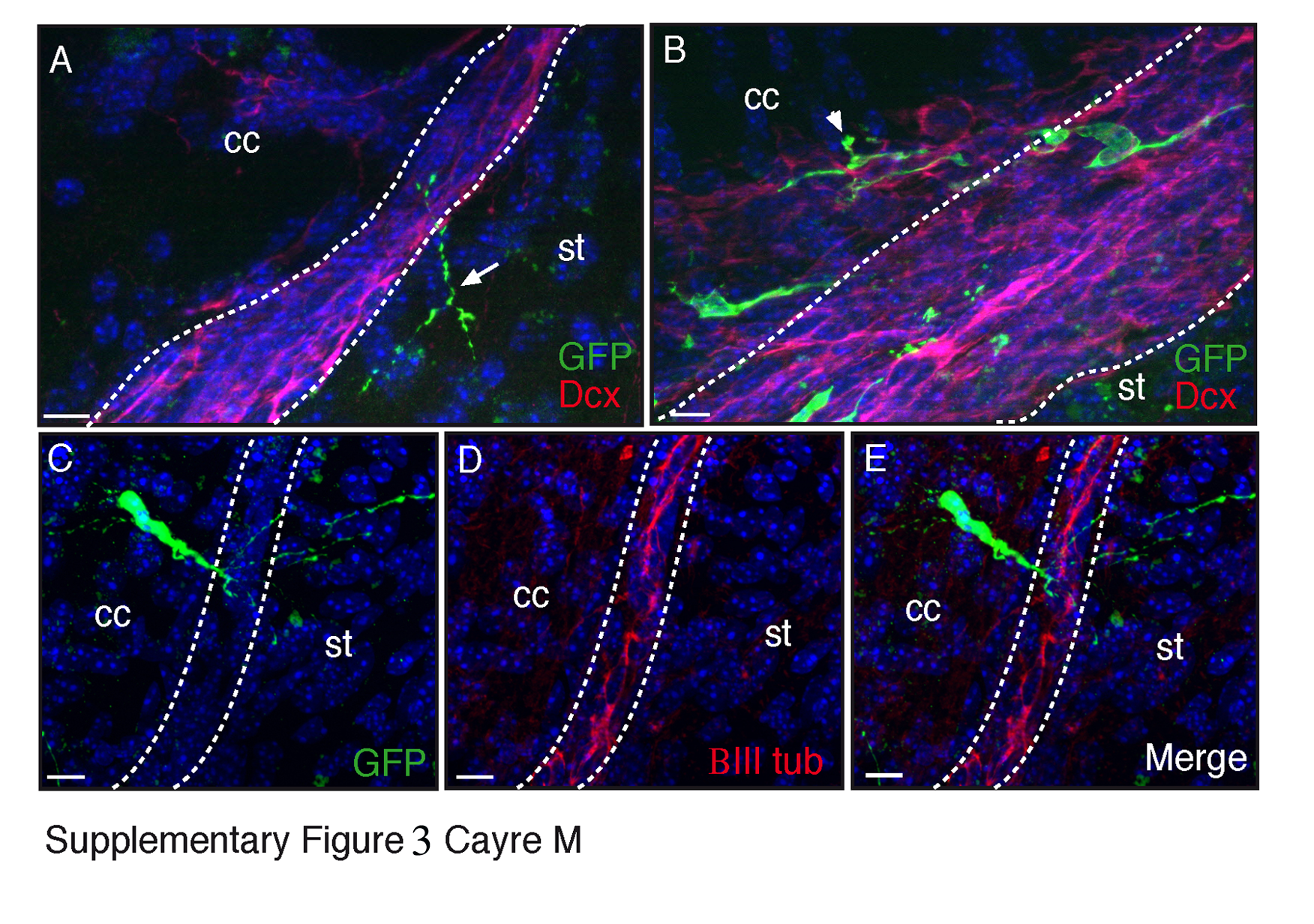

Supplement: Figure S3 — In TgRln mice, cells that leave the RMS migrate either individually or in chains, indifferently toward the striatum or the corpus callosum, and exhibit complex morphology. A–B: Immunolabeling of GFP (grafted SVZ cells, in green) and DCX (endogenous neuroblasts, in red) in TgRln mice, showing that GFP+ grafted cells that escape from the RMS can orientate either toward the corpus callosum (cc) or toward the striatum (St). Some of them migrate individually by detaching from other progenitors (arrow in A) while others escape following DCX positive chains that are derived from the stream (arrowhead in B). Note the particularly mature morphology of detached grafted cells (arrow in A). C–E: Illustration of a grafted cell (GFP-positive) that escape the RMS in a TgRln mouse, and loose ßIII tubulin expression (in red). Scale bars = 10 µm. (TIFF) [file pone.0020430.s003.tif]

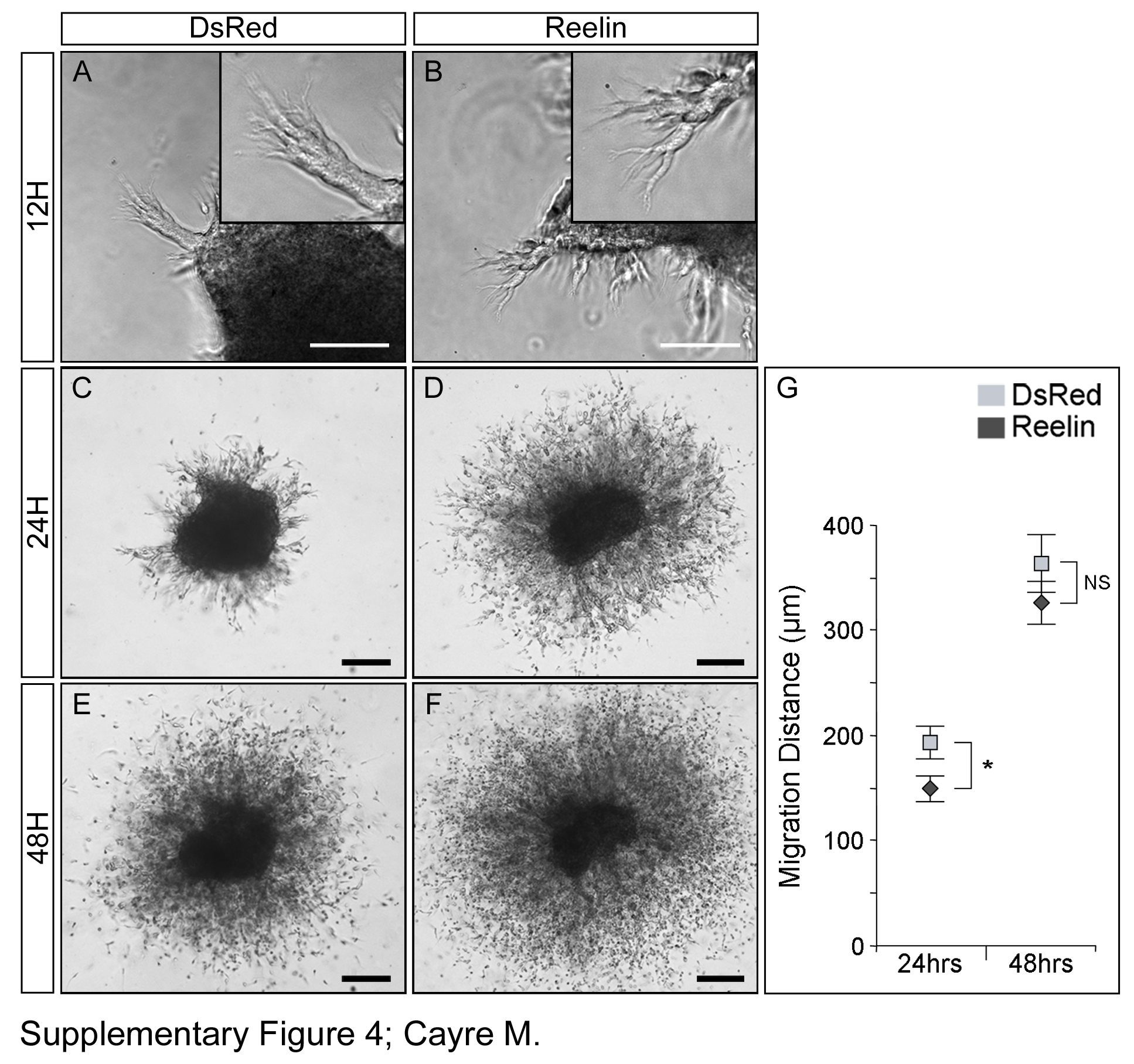

Supplement: Figure S4 — Dynamic analysis of cell migration from SVZ explants in absence and presence of Reelin. Illustrations at 12 hours (A), 24 hours (B) and 48 hours (C) after plating. Note that pictures in C–D and E–F show the same explants at 24 and 48 hours. A,B: Effect of Reelin on early steps of cell migration from SVZ explants in vitro. The first signs of cell migration can be observed 12 hours after plating. Even in presence of Reelin in the culture medium, cells emerge from the explants performing chain migration. No obvious difference in chain length can be observed at that time between the two conditions. However, chains appear to be slightly less compacted in presence of Reelin (see magnification in boxes). C, D: 24 hours after plating, both cell detachment and chain length are notably increased in presence of Reelin. E, F: At longer time point (48 hours), the effect of Reelin on chain length is no longer significant. Cell detachment from the chains is becoming visible in control condition but it is still more obvious in presence of Reelin. G: Quantification of migration distance at 24 and 48 hours in absence and presence of Reelin. Scale bar: A, B = 50µm; C–F = 100µm. (TIFF) [file pone.0020430.s004.tif]

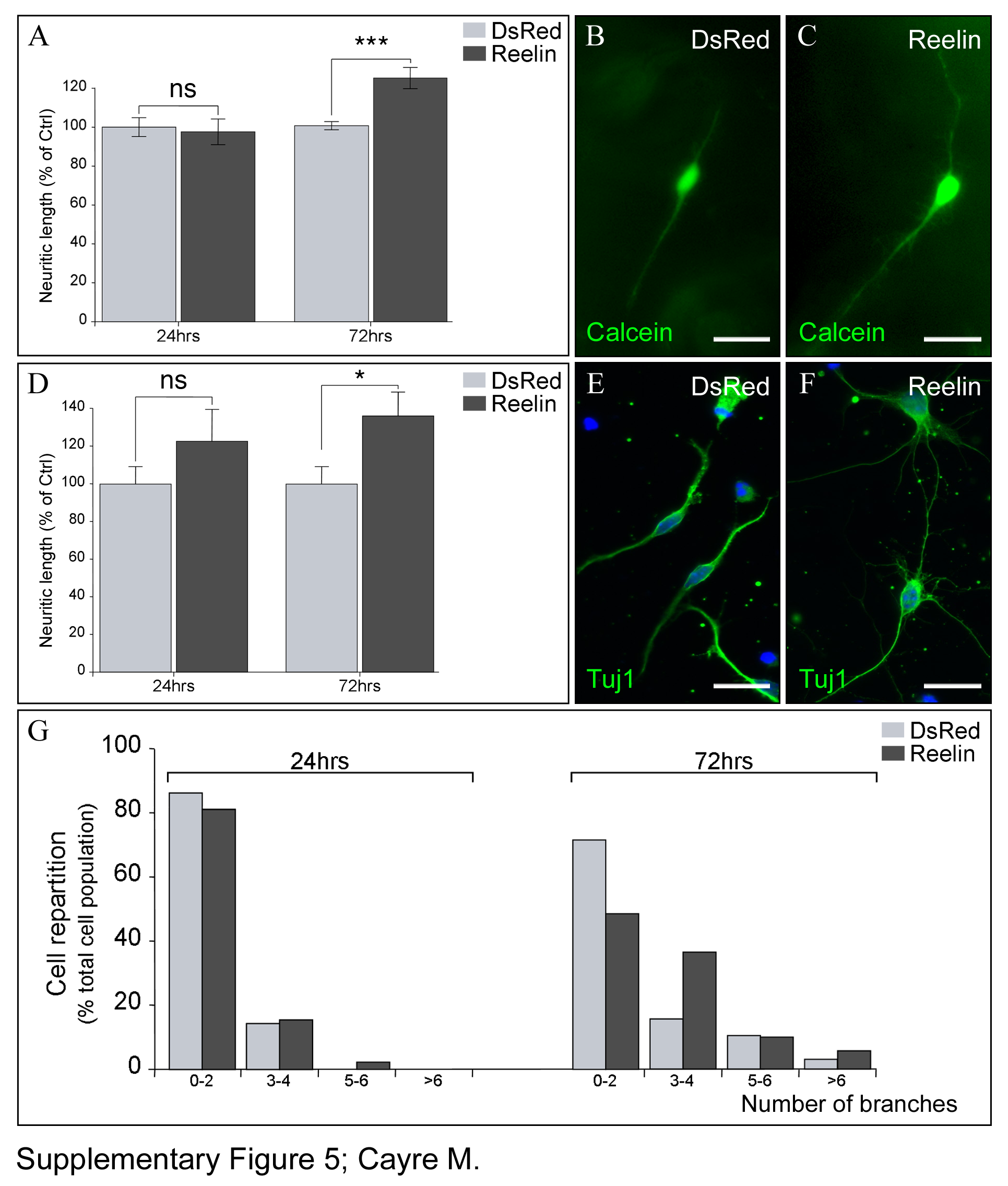

Supplement: Figure S5 — Effect of Reelin on SVZ-derived neuronal progenitor maturation in vitro . A–F: effect of Reelin on total neurite length, either in pro-migratory conditions (explant cultures (A–C)) or in differentiation conditions (dissociated cell culture (D–F)) 24 and 72 hours after plating. Neurite length is not affected at 24 hours but is increased in presence of Reelin after 72 hours. B,C and E,F: Illustration of typical morphology of SVZ-derived progenitors in absence (B,E) or presence (C,F) of Reelin, after 72 hours of culture. G: Complexity of neuritic arborisation of SVZ-derived Tuj1-positive progenitors after 24 hours and 72 hours of culture. The presence of Reelin in the culture medium increases the number of branches per neuron at 72 hours but not at 24 hours. Scale bar: B–C = 20µm; E–F = 20 µm. (TIFF) [file pone.0020430.s005.tif]

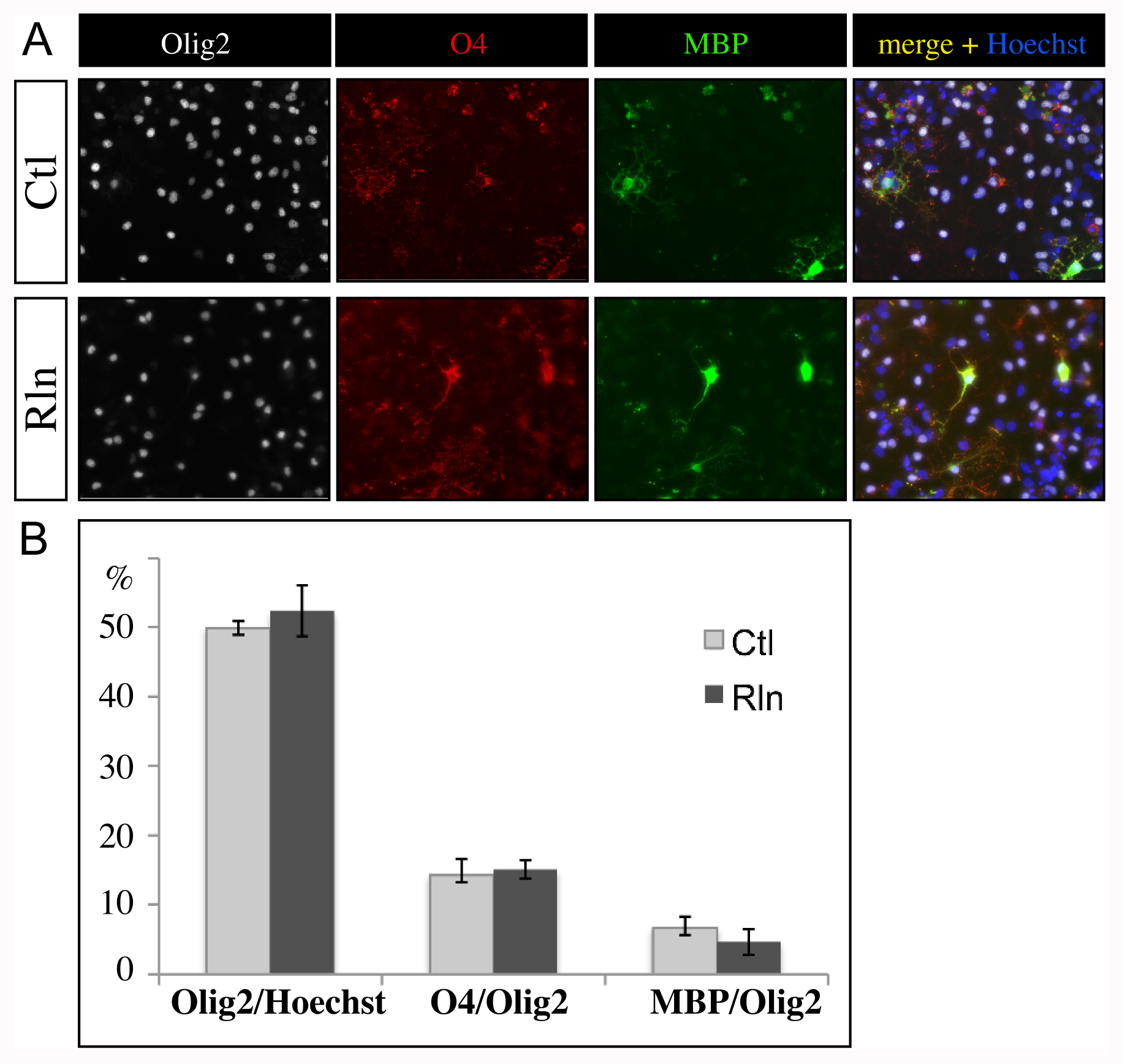

Supplement: Figure S6 — Effect of Reelin on SVZ-derived oligodendrocyte progenitor maturation in vitro . A: Triple Olig2/O4/MBP immunolabeling of SVZ-derived neurospheres after 3 days differentiation in vitro, in presence (Rln) or absence (Ctl) of Reelin. B: quantification shows no effect of Reelin on the oligodendroglial fate or on the maturation of the progenitors. (TIFF) [file pone.0020430.s006.tif]

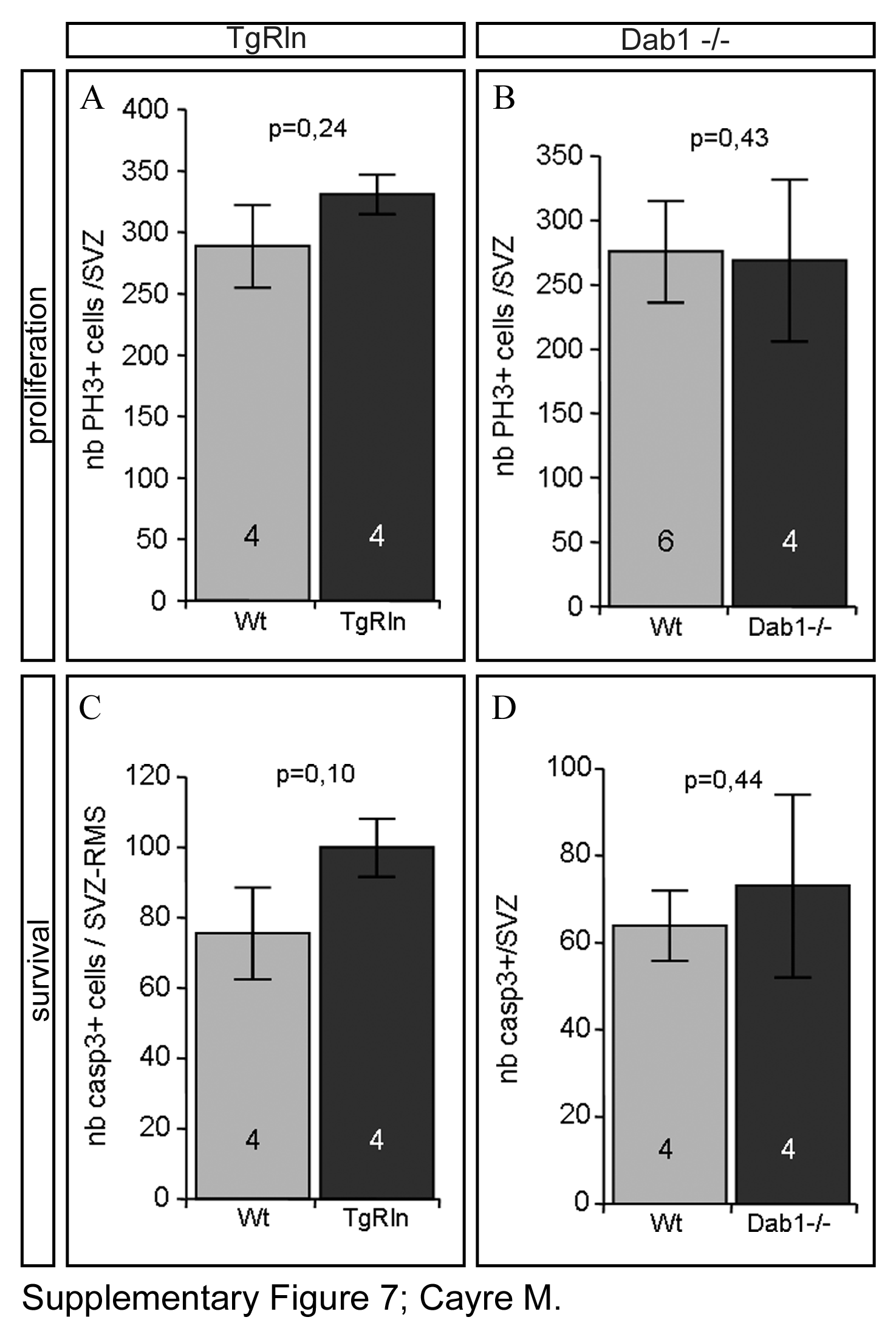

Supplement: Figure S7 — SVZ cell proliferation and survival in TgRln and Dab1−/− mice with LPC lesion in the corpus callosum as compared to their wild type littermates. No significant difference could be detected in the number of PH 3 or casp3-positive cells between wild type and transgenic mice. The number of mice analyzed is indicated inside each column, and the significance (Mann Whitney) indicated above each graph. (TIFF) [file pone.0020430.s007.tif]

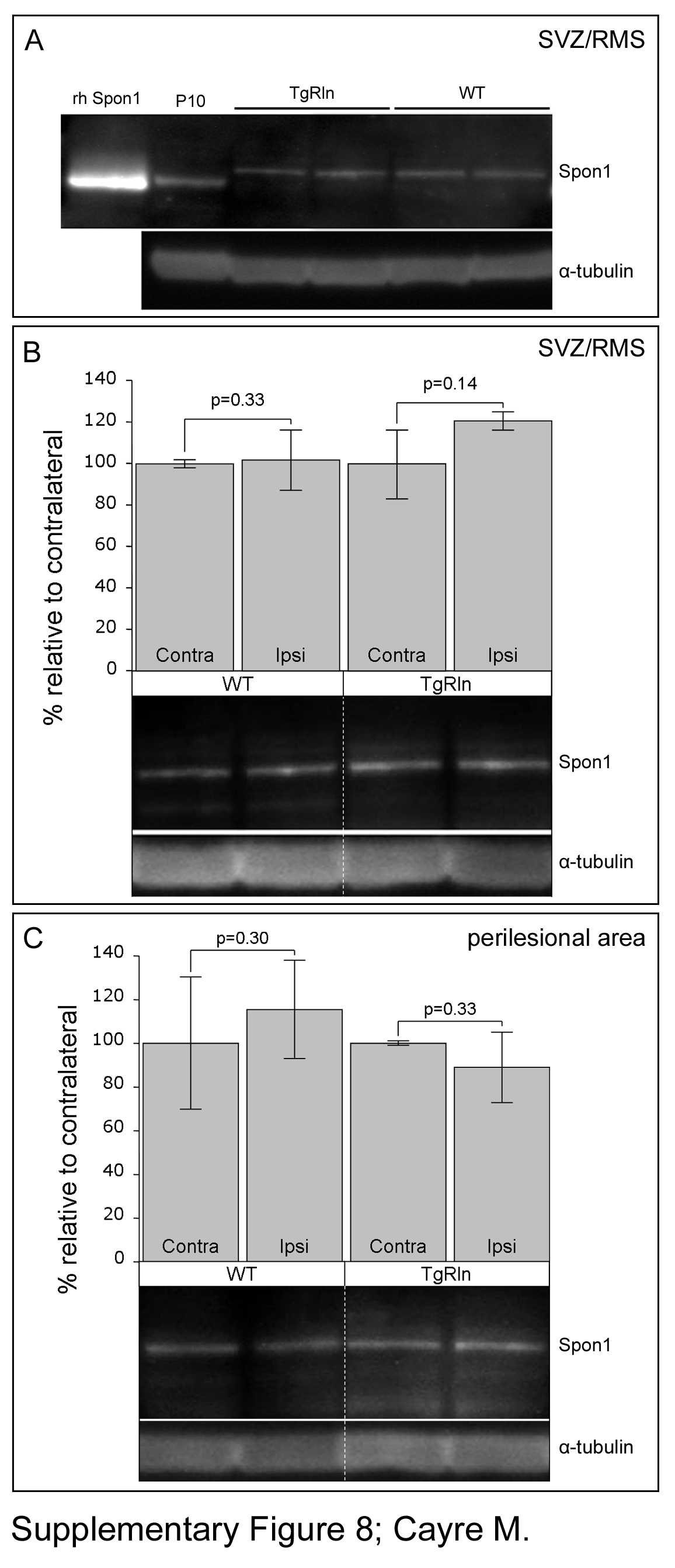

Supplement: Figure S8 — F-spondin expression in healthy and demyelinated (LPC) wild-type and transgenic mice overexpressing Reelin. A: illustration of a western blot showing F-spondin expression in physiological conditions, in the SVZ/RMS of neonates (pool of 6 ten day-old mice) and adult mice (2 transgenic and 2 wild type mice). Recombinant human F-spondin (Spon1) was used as a positive control. F-spondin expression is low in adults but similar in Wt and TgRln mice. B–C: after LPC lesion, F-spondin expression is not significantly increased in SVZ/RMS (B) nor in perilesional structures (C) in Wt (n = 2) and TgRln mice (n = 3). F-spondin expression has been normalized to α-tubulin and expressed in percent of unlesioned side (contralateral). (TIFF) [file pone.0020430.s008.tif]
